# Landscape-wide cosmogram built by the early community of Aguada Fénix in southeastern Mesoamerica

**DOI:** 10.1126/sciadv.aea2037

**Published:** 2025-11-05

**Authors:** Takeshi Inomata, Daniela Triadan, Verónica A. Vázquez López, Melina García Hernández, Juan Carlos Fernandez-Diaz, Ashley E. Sharpe, Claudia Alvarado, Atasta Flores, Xanti Ceballos, Kelsey E. Hanson, Ran Chen, Timothy Beach, Takayuki Omori, Hiroo Nasu, Kazuo Aoyama, Keitaro Yamada, Ikuko Kitaba, Takeshi Nakagawa

**Affiliations:** ^1^ School of Anthropology, University of Arizona, Tucson, AZ 85721, USA.; ^2^ Institute of Archaeology, University College London, London WC1H 0PY, UK.; ^3^ Middle Usumacinta Archaeological Project, Balancán 86850, Mexico.; ^4^ National Center for Airborne Laser Mapping (NCALM), University of Houston, Houston, TX 77204-5059, USA.; ^5^ Center for Tropical Paleoecology and Archaeology, Smithsonian Tropical Research Institute, Balboa 0843-03092, Republic of Panama.; ^6^ Colegio de Morelos, Cuernavaca 67050, Mexico.; ^7^ Department of Sociology and Anthropology, University of Texas at Arlington, Arlington, TX 76019, USA.; ^8^ Department of Geography & the Environment, University of Texas, Austin, TX 78712, USA.; ^9^ University Museum, University of Tokyo, Tokyo 113-0033, Japan.; ^10^ Center for Fundamental Education, Okayama University of Science, Okayama 700-0005, Japan.; ^11^ Faculty of Humanities, Ibaraki University, Mito 310-8512, Japan.; ^12^ Faculty of Science, Yamagata University, Yamagata 990-8560, Japan.; ^13^ Research Centre for Palaeoclimatology, Ritsumeikan University, Shiga 525-8577, Japan.

## Abstract

There is growing recognition that societies without prominent hierarchies could build large constructions. Scholars are debating what motivated many people to participate in these construction projects. We investigated the site of Aguada Fénix, Mexico, which features the oldest and largest monumental architecture in the Maya area. Using light detection and ranging (LiDAR) and excavations, we documented a site plan composed of nested cross forms built between 1050 and 700 BCE. Its center was marked by a large cruciform cache containing the earliest known directional color symbols in Mesoamerica. The overall pattern consisted of 9- and 7.5-kilometer-long axes delineated by canals and corridors. The builders constructed canals, measuring up to 35 meters wide and 5 meters deep, and a dam to supply them with lake water. Although the canals appear unfinished, this site plan exceeded or rivaled the extents of later Mesoamerican cities. Aguada Fénix was probably designed as a cosmogram, which likely attracted people from a broad area.

## Teaser

The cosmogram of Aguada Fénix built over the landscape between 1050 and 700 BCE rivaled the extents of later Mesoamerican cities.

## INTRODUCTION

With the better understanding of monumental sites from preagricultural or initial agricultural periods in various parts of the world, a growing number of researchers recognize that large buildings could be constructed without prominent social hierarchies (*1*–*7*). In the Maya area, however, such early monumental constructions had not been recognized until recently. Scholars had long assumed that the inhabitants began adopting ceramics and built small villages during the early part of the Middle Preclassic period around 1000 to 700 BCE and that large centers did not develop until the Late and Terminal Preclassic periods (350 BCE to 250 CE) (*8*, *9*). This view began to be questioned with the finds of large constructions at early Middle Preclassic sites, such as Ceibal, Cival, Yaxnohcah, and Xocnaceh (*10*–*17*). In particular, the discovery of Aguada Fénix with a large artificial plateau (Main Plateau) built during the early Middle Preclassic period generated scholarly debate about the development of early Mesoamerican civilizations. In contrast to the Olmec centers of San Lorenzo and La Venta, those Middle Preclassic sites of the Maya area did not show evidence of prominent social hierarchies. What motivated people to participate in large construction projects, and how did they achieve them in the absence of powerful elites? These questions have far-reaching implications for our understanding of social systems, including the contemporary issue of how we build large-scale organizations while suppressing excessive inequality.

We addressed these questions by examining the design and functions of Aguada Fénix. The results showed that the plan of Aguada Fénix consisted of nested cross patterns, which likely formed a cosmogram representing worldviews and calendrical concepts. The largest cross form, extending across the landscape, rivaled or exceeded the extents of later Mesoamerican cities, such as Tikal and Teotihuacan. It included a large-scale hydraulic system, consisting of a dam and canals, measuring up to 35 m in width and 5 m in depth. Although the canals were not completed before the abandonment of the site, the scale of the overall design and construction of Aguada Fénix are more ambitious and impressive than our initial assessment based mainly on the size of the Main Plateau. Along with the appeals of collective ceremonies, feasting, and the exchange of goods, the construction of a cosmogram, materializing the order of the universe, likely provided a rationale for a large number of people to participate without coercive force. The development of Aguada Fénix exemplifies the capabilities of human organization without prominent inequality, but it also hints at the challenges that earlier builders faced.

### Setting

Aguada Fénix is located in southeastern Mexico, in the southwestern part of the area conventionally called the Maya lowlands. Although we do not assume that the builders of Aguada Fénix were Mayan language speakers, we recognize their strong cultural continuity to the later Maya communities. Our initial excavations showed that the ceramic use at this site started around 1200 BCE and the Main Plateau, measuring 1400 m by 400 m horizontally and 10 to 15 m vertically, was constructed and renovated between 1050 and 700 BCE (*18*). This makes the Main Plateau the largest and oldest monumental construction known in the Maya area. The lack of evidence for substantial social inequality suggests that the builders of Aguada Fénix did not have prominent social hierarchies. In addition, we have not found permanent residences and suspect that a considerable portion of them retained some level of residential mobility.

The central precinct of the Main Plateau was a complex consisting of two western mounds and an elongated eastern platform. This arrangement is generally called the E Group, which was built during the Middle Preclassic and later periods at numerous sites across the Maya lowlands, Chiapas, and the southern Gulf Coast region, as focal points of community ritual (*15*, *19*–*25*). The E Group at Aguada Fénix was possibly aligned with the sunrise on 24 February and 17 October with an interval of 130 days, that is, half of the 260-day cycle of the Mesoamerican ritual calendar (*26*, *27*).

**Fig. 1.**
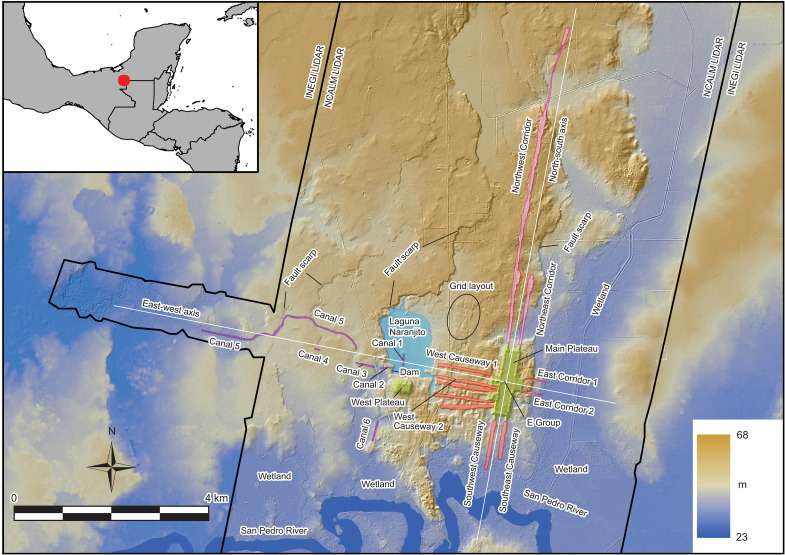
Map of Aguada Fénix. The map is based on LiDAR data obtained by the National Center for Airborne Laser Mapping (NCALM) and the Instituto Nacional de Estadística y Geografía (INEGI). The white lines indicate the north-south and east-west axes of the site. Plateaus, causeways, corridors, canals, and the laguna are shown in different colors.

Although E Groups were common at other sites, Aguada Fénix, along with the smaller and later site of Nixtun-Ch’ich’ (*28*, *29*), was unique in that the east-west and north-south axes extended over a large area (Fig. 1). In each of the four directions, a pair of causeways (raised above the ground level) and corridors (cut into the ground) radiated from the plateau, running parallel to the axes. The longest of these, the Northwest Corridor, measured 6.3 km. West Causeways 1 and 2 ended at Laguna Naranjito, but the axis may have extended farther to the west. On the southwestern shore of the laguna was a dam, which probably served to control the water level of the lake. Farther west, the light detection and ranging (LiDAR) image showed possible canals (Canals 1 to 6), most of which appeared to run parallel to the axes. Canals 3 and 4, however, were short, and their functions and dates needed to be examined.

From 2020 to 2024, we examined the presence of a site plan on the basis of those axes and its social implications by investigating the dates and functions of its main components. This involved the intensive excavation of the E Group Plaza, coring Laguna Naranjito, and analyzing the dam and the canals through an additional LiDAR survey, excavations, and auger tests.

## RESULTS

### Ritual deposits in the E Group Plaza

**Fig. 2.**
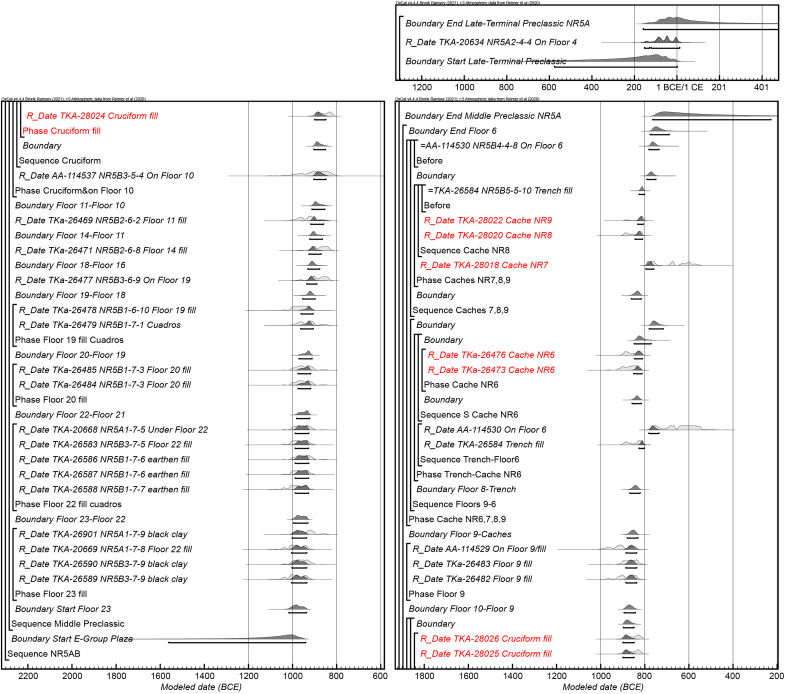
Bayesian modeled radiocarbon dates from Op. NR5. Outliers have been removed. Dates associated with caches are marked in red (see data S1 and S2).

Our previous excavations and auger tests showed that the builders first focused their efforts to build up the northern and southern sections of the Main Plateau over lower parts of the natural terrain around 1050 BCE. In the E Group Plaza, our excavation [Operation (Op.) NR5] revealed a series of floor buildups reaching a total fill thickness of 2.5 m. The earliest construction placed on limestone bedrock (Floor 23 fill) in this area dates to 1020–935 BCE with the highest probability (HP) around 980 BCE (all date ranges are given in calibrated dates at 95.4% probability after Bayesian analysis) (Fig. 2, Supplementary Text, and data S1 and S2). By 915–850 BCE, when the builders constructed Floor 10a in the E Group Plaza, they had accumulated a total fill thickness of 1.6 to 1.8 m.

**Fig. 3.**
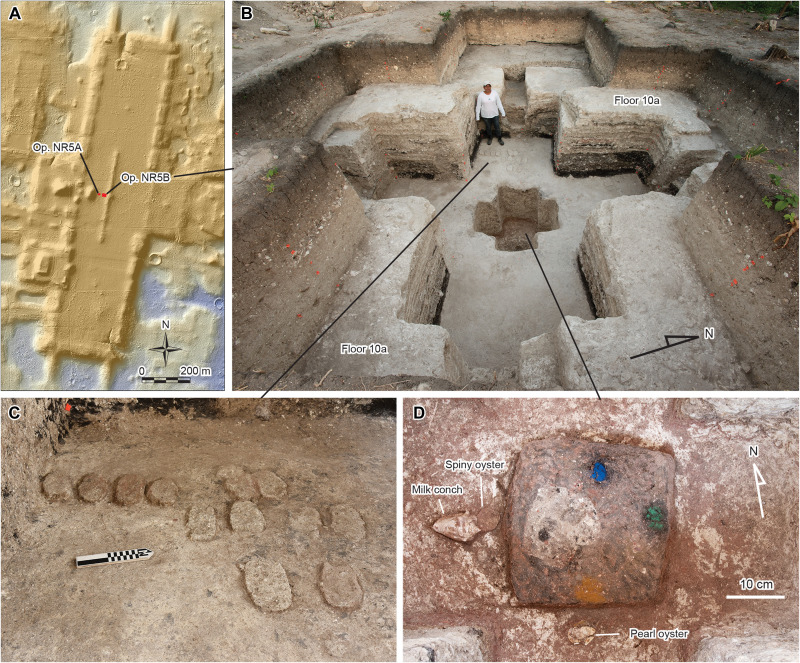
Cruciform cache found in the E Group Plaza. (**A**) Locations of the excavation units in the E Group on the Main Plateau. (**B**) Cruciform cache viewed from the east. (**C**) Axe-shaped clay objects found at the bottom of the large cruciform pit (Cache NR10). (**D**) Pigments and shells found at the bottom of the small cruciform pit (Cache NR11).

**Fig. 4.**
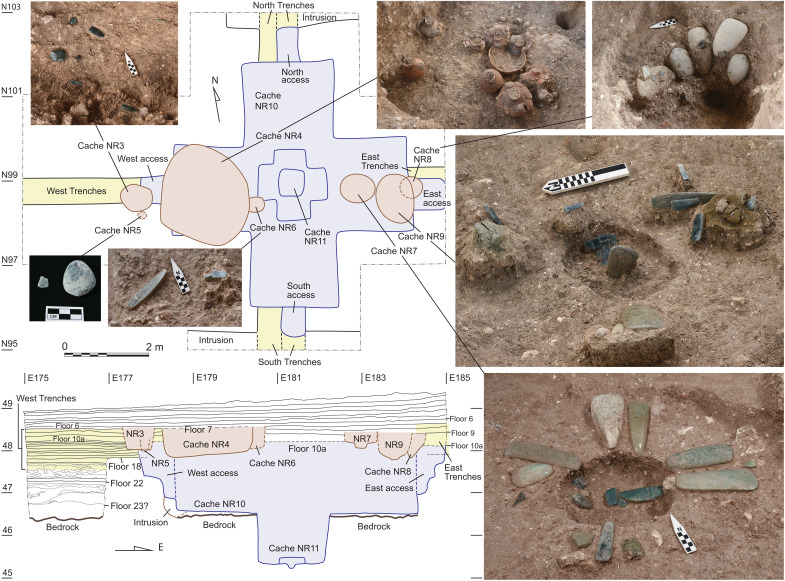
Locations of Caches NR3-9 shown in plan view and profile of the cruciform pit.

**Fig. 5.**
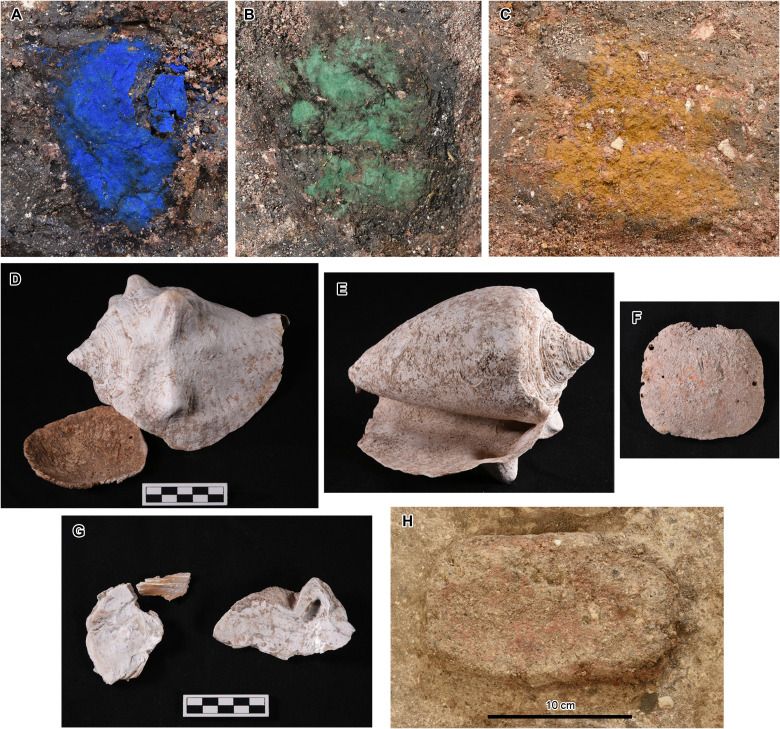
Cache NR10/11 found in the cruciform pit. (**A**) Blue pigment (azurite) found in the northern part of Cache NR11. (**B**) Green pigment (malachite) found in the eastern part. (**C**) Yellow pigment (ochre containing goethite) found in the southern part. (**D**) Atlantic milk conch (*M. costatus*) and a valve of the marine spiny oyster (*Spondylus* sp.) found in the western part. The outer lip of the conch was partially cut clean, and the exterior surface of the spiny oyster was smoothed. The edges of the spiny oyster were also removed to form a square shape, and the valve contained at least two purposefully drilled holes, perhaps for use as a pendant. (**E**) Atlantic milk conch underside. (**F**) Marine spiny oyster exterior. (**G**) Valve of a marine pearl oyster (*Pinctada* sp.) found in the southern part. The exterior surface of this oyster was also smoothed. (**H**) Close-up view of one of the axe-shaped clay objects with red pigment found in Cache NR10.

The builders cut a cruciform pit into Floor 10a along the east-west axis of the E Group. It measured 5.9 m north-south and 5.6 m east-west, dug down to the bedrock surface (Figs. 3 and 4). At the bottom of this pit in its center was a smaller cruciform pit dug into the limestone bedrock to a depth of 1.1 m. The large cruciform pit had a narrow, stepped accessway cut into each of its four sides, through which ritual practitioners could enter the interior of the pit. The walls of the pit, including those of the accessways, were covered by a thin layer of white clay, suggesting that the pit was kept open for some time. At the bottom of the large pit, we found 24 axe-shaped objects made of unbaked clay, some with traces of red pigment, probably made of ochre containing hematite (Cache NR10) (Fig. 5). There may have been more axe-shaped objects made of unbaked clay besides the 24 that we identified. They may have been too compressed or indistinguishable from the surrounding fill materials.

The small cruciform pit also contained a ritual deposit at the bottom (Cache NR11) (Fig. 5). Although we use two separate designations, Caches NR10 and NR11 made up a single ritual feature used and buried at the same time. At the bottom of the small pit, the builders made a 0.10-m-deep depression in the limestone bedrock, in which they appear to have placed a wooden post. The post was then supported by a square lump of black clay. On this lump, they placed blue pigment made of azurite [Cu_3_(OH)_2_(CO_3_)_2_] in the northern part, green pigment made of malachite [Cu_2_CO_3_(OH)_2_] in the east, and yellow pigment made of ochre containing goethite [FeO(OH)] in the south (Fig. 5, Supplementary Text, and table S1). The western part was a hollow filled with the same soil as the surrounding fill, and there may have been an object made of organic materials.

To the south of the clay lump was a valve of a marine pearl oyster (*Pinctada* sp.), and to the west were an Atlantic milk conch (*Macrostrombus costatus*) and a valve of marine spiny oyster (*Spondylus* sp.) (Figs. 3 and 5 and Supplementary Text). These shells may also have represented directional colors as the milk conch and *Spondylus* originally showed red colors, and the pearl oyster may have had a faint golden/yellow cast. Moreover, these shells may have symbolized water tied to Laguna Naranjito and the San Pedro River, as well as the Gulf of Mexico and the Pacific Ocean beyond them. The milk conch placed on the western side is an Atlantic species, likely having come from the Gulf of Mexico in this case. The exact species of *Spondylus*, also placed on the western side, could not be identified because its edges and hinge had been purposefully cut away. Thus, it could have come from either Atlantic or Pacific oceans. The origin of the pearl oyster valve, placed on the southern side, could not be identified with certainty because of the physical similarities between the Atlantic and Pacific species of this genus (*P. margaritifera* and *P. mazaltanica*, respectively).

We did not find any objects on the northern and eastern sides of the black clay lump, but there may have been offerings made of perishable material. Phytolith analysis indicated that soils on all four sides of the black clay lump contained high frequencies of epidermal cells (Supplementary Text, fig. S1, table S2, and data S3). Although they do not point to specific plant species, their abundance suggests the presence of plant matter, which may have been used as offerings or materials for wrapping or covering objects.

### Cache closing and later offerings

Before filling the pit, the builders covered Cache NR11 with a thin layer of reddish sand containing iron oxide and quartz grains, which appeared similar to the alluvial deposits of the Usumacinta River, located 30 km west of the site. They also placed a thin layer of the same reddish sand at the bottom of the large pit, covering the axe-shaped clay objects of Cache NR10. The large and small pits were then filled with a mixture of various types of soil, including black clay and whitish, marl-like material. Radiocarbon dates indicate that Cache NR10/11 was made in 900–845 BCE (HP 880 BCE) (Fig. 2). Although Cache NR10/11 is the earliest confirmed ritual deposit found in the E Group Plaza thus far, the intrusion cut from Floor 22 found on the western edge of Cache NR10 may have contained offerings made of perishable materials (Fig. 4). Thus, there may be other ritual deposits predating Cache NR10/11 in the E Group Plaza.

**Fig. 6.**
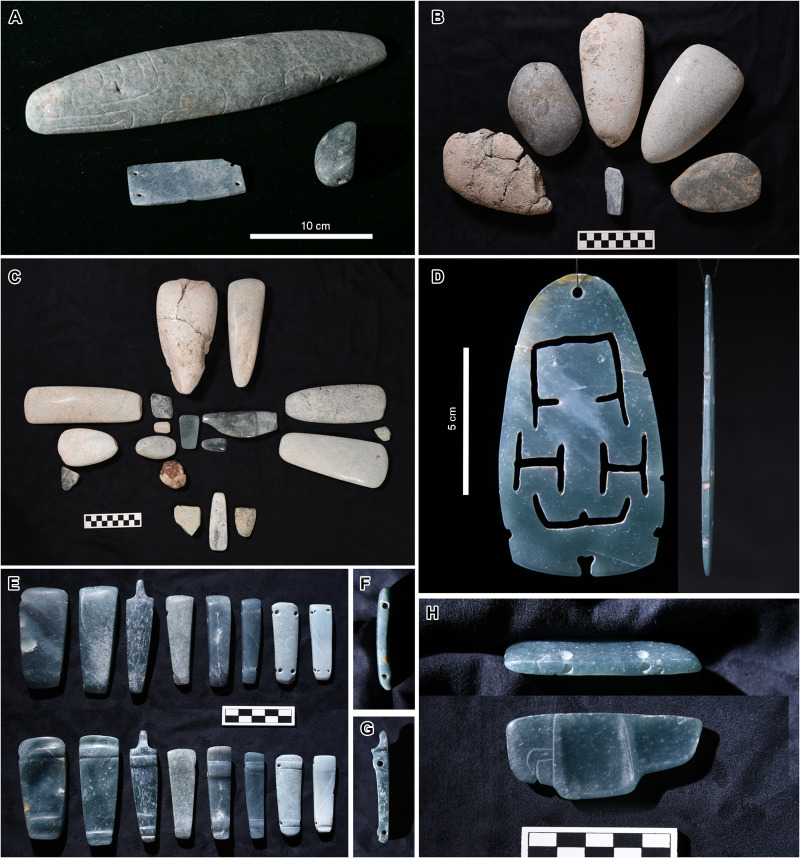
Greenstone objects found in Caches NR6-9. (**A**) Cache NR6. The large incised object probably depicts a crocodile. (**B**) Cache NR8. It contained five greenstone axes and an ornament arranged in a flower-petal form but probably had more objects before it was cut by Cache NR9. (**C**) All objects found in Cache NR7. The cache contained 18 stone objects placed in a cruciform pattern. (**D**) Front and side view of a thin jade plaque found in the central part of Cache NR9. It may have been used as a plaque hanging from a belt or as a pendant. It probably represents a female in a birthing position. It appears to have broken when the upper right notch was being made. (**E**) Two sides of the jade objects, each with two transverse perforations, found in Cache NR9. They may have been used together as a headband or necklace. (**F**) Side view of the first object from the left in (E). (**G**) Side view of the third object from the left in (E). (**H**) Pectoral probably representing a bird found in the central part of Cache NR9.

After they filled Cache NR10/11, people probably remembered this area as an important location and continued to place ritual deposits, containing greenstone objects and ceramic vessels, between 855 and 760 BCE (Caches NR3-9) (fig. S2). Greenstone ornaments included representations of a crocodile, bird, and possibly a human female in a birthing position (Fig. 6). In Caches NR7 and NR9, the objects were arranged in a cruciform pattern. We also found trenches extending from the large cruciform pit along its axes (Fig. 4). The builders dug these trenches multiple times roughly contemporaneously with Caches NR3-9 after the cruciform pit was filled. Cache NR10/11, along with the later caches and the trenches, represented directional symbolism and marked the center of the landscape-wide cross form.

### Laguna Naranjito Dam

Laguna Naranjito is a shallow body of water formed in a west-tilted half-graben. Today, Laguna Naranjito is mostly dry, with shallow water remaining only in its southwestern part, but the local people have told us that the lake held deeper water several decades ago. Our coring recovered 3.65-m-deep sediments consisting mostly of gray clay over bedrock. Four radiocarbon samples from various depths returned dates after 1950 CE, indicating that eroded soils filled the lake after modern deforestation (table S3).

**Fig. 7.**
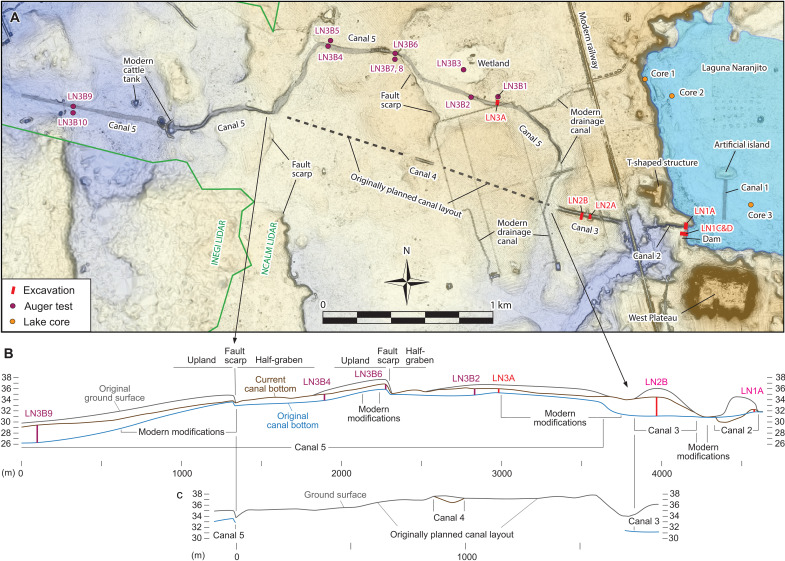
Dam and canals. (**A**) Locations of the excavations and auger tests. Canals are lightly shaded. (**B**) Section drawings of Canals 2 to 5 showing the estimated original ground surfaces before canal construction, the current canal bottoms, and the original canal bottoms before the canals were filled with sediments.

**Fig. 8.**
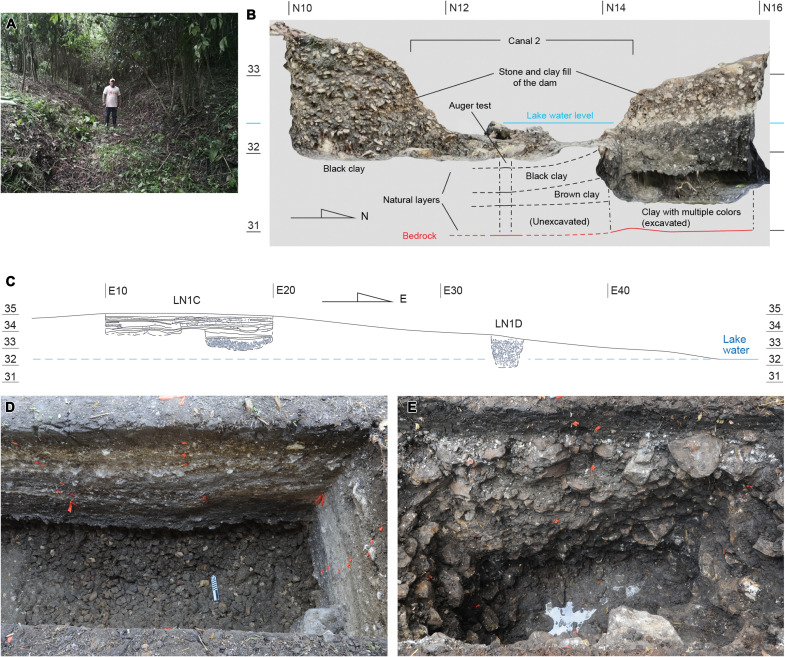
Excavation of the dam (Op. LN1). (**A**) Dam and Canal 2 viewed from the east. (**B**) North-south cross section of the dam and Canal 2 where a gate may have existed (Op. LN1A). Note that the dam was constructed of stones and compact black clay placed over natural soil. The current lake water level during the dry season is indicated in light blue. (**C**) East-west cross section of the dam along Ops. LN1C and LN1D. Op. LN1C did not reach bedrock because the excavation unit became unstable upon reaching the fill of loose stones. In Op. LN1D, we encountered a fill made of stones larger than those found in Ops. LN1A and LN1C, mixed with compact black soil. We halted the excavation before reaching bedrock due to the difficulty caused by the presence of many stones. Note that the stone-clay fill in Op. LN1D extended below the current dry-season water level. These findings indicate that the builders first placed loose stones in the area around Op. LN1C and then added a layer of stones mixed with compact black clay to create an impermeable dam. Subsequently, they constructed Structure Chejé with a series of floors atop the dam. (**D**) Eastern part of Op. LN1C viewed from the south. The layer of loose stones is visible at the bottom. (**E**) Op. LN1D viewed from the south.

The dam built on the southwestern shore of the laguna measured 120 m in length and 30 to 55 m in width (Fig. 7 and fig. S3). During the rainy season today, water flows out of the laguna through the point where Canal 2 cuts through the northern end of the dam. At this cut, we saw several stone blocks, which may have supported a wooden gate to control the water level. We placed a trench (Op. LN1A) across this point, but it was unclear whether a gate had ever been present (Fig. 8). This excavation revealed that the fill of the dam consisted of exhausted chert flake cores and hard dolomite or crystalline limestone cobbles densely compacted with black clay. It was nearly devoid of ceramics and bones. The results of the phytolith analysis suggested that the black clay used in the fill was probably taken from the laguna or nearby wetlands (table S2 and data S3). The fill was placed on natural soil, found at an elevation 20 cm below the current dry-season lake water level. Another excavation conducted near the center of the dam (Op. LN1C) uncovered a probable structure (Structure Chejé) built on the dam and associated with middens (Fig. 8 and fig. S4). The middens contained ceramics dating to the early Middle Preclassic period, and seven radiocarbon dates fell between 930 and 820 BCE (figs. S5 and S6 and data S1). The dam was probably built some time before these midden dates, contemporaneously with the Main Plateau.

### Canals

**Fig. 9.**
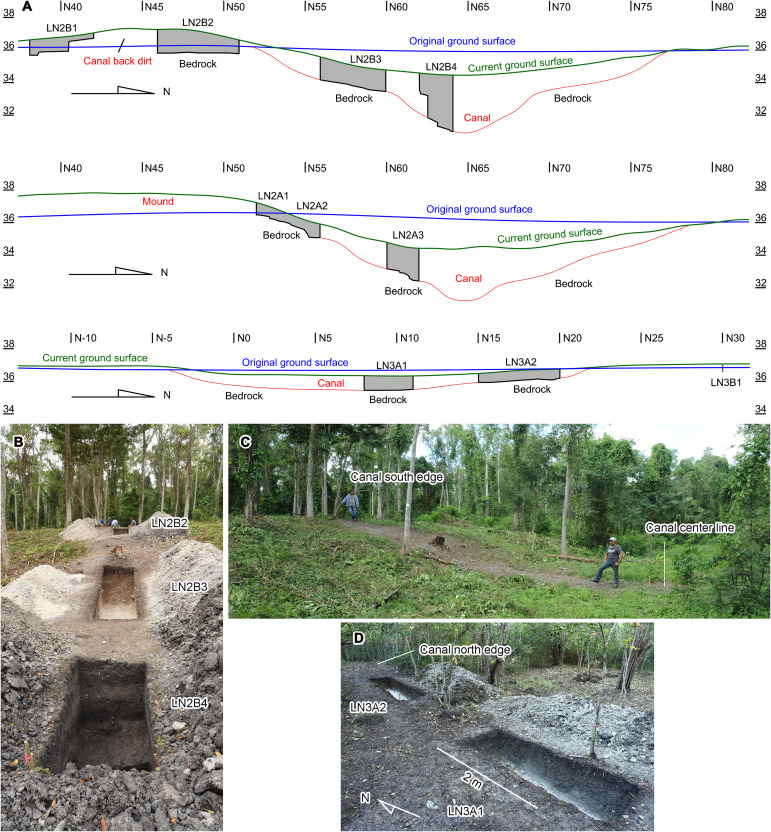
Excavations of Canal 3 (Op. LN2) and Canal 5 (Op. LN3). (**A**) North-south cross sections of Canal 3 (Ops. LN2B and LN2A) and Canal 5 (Op. LN3A). Estimated original ground surfaces are indicated, along with current ground surfaces and canal bottoms. (**B**) Op. LN2B viewed from the north. (**C**) Area of Op. LN2B over Canal 3 before excavation, viewed from the east. (**D**) Op. LN3A viewed from the southwest.

The terrain to the west of Laguna Naranjito is defined by mostly horizontal bedrock of limestone and marl, which was covered by shallow soil and modified by a series of 1.5- to 2.0-m-high fault scarps, west-tilted half-grabens, and uplands. Canals 3, 4, and 5 extended across this area, which the local people told us were not modern constructions (Fig. 7 and fig. S3). Canal 3 measured 420 m long and 25 to 30 m wide. LiDAR data and our ground survey suggested that the builders piled a substantial amount of back dirt from the digging of Canal 3 along its southern edge and a smaller amount along the northern edge. A possible prehispanic mound was built on the canal back dirt on the southern edge (fig. S3). We excavated two trenches across Canal 3, one over the mound (Op. LN2A) and the other to the west of it (Op. LN2B) (Figs. 7 and 9 and fig. S3). Ceramic data indicated that the mound was constructed during the Late Classic period (600 to 900 CE), which confirmed that the canal was built in prehispanic times. To construct the canal, the builders dug through natural marl and limestone to an approximate depth of 5 m below the original ground surface. The canal bottom was 1.3 m lower than the current water level of Laguna Naranjito during the dry season.

Farther west, Canal 4, measuring 120 m in length and 18 m in width, cut through the highest point near the center of the upland (Fig. 7 and fig. S3). We suspect that the builders originally intended to construct a straight canal connecting Canals 2, 3, and 4 and the western section of Canal 5. By digging Canal 4 at the highest point near the center of the upland, they may have tried to open a sight line before extending Canal 3 to the west. Constructing a straight canal would have required cutting through the upland between Canals 3 and 4, which the builders appear to have determined unpractical. They changed the plan and began to dig Canal 5 circumventing the upland. Our LiDAR data obtained in 2023 showed that Canal 5 measured 3650 to 4200 m in length and 15 to 35 m in width. We conducted one trench excavation (Op. LN3A) and 10 auger tests (Op. LN3B) along this canal (Fig. 7 and fig. S3). The results suggested that the canal was dug 1.3 to 3.3 m deep from the original ground surface. The highest point of the canal bottom was found in Auger LN3B6, placed above a fault scarp. This point was 5.4 m higher than the bottom of Canal 3 (Fig. 7 and table S4). From there, Canal 5 slopes down gradually both to the east and west. There is a small wetland to the north of Canal 5, but it is not connected to the canal and does not hold sufficient water to fill the canal. Nor would the water taken from Laguna Naranjito reach beyond Canal 3. In other words, Canal 5 was never completed.

### Construction date and function

The floatation of soil samples from the excavations and auger tests of Canals 3 and 5 did not yield any charcoal adequate for radiocarbon dating. We also attempted to extract pollen for radiocarbon dating from soil, using a cell sorter (*30*, *31*), but only a small quantity of pollen was present (Supplementary Text, fig. S7, and tables S5). However, the ceramics provided important information about when the canals were constructed and how sedimentation processes occurred. We found ceramics, dating to 1200 to 700 BCE, such as Huetche White, at the bottom of Canals 3 and 5 directly on bedrock (figs. S6, S8, and S9). The back dirt of Canal 3 excavated in Op. LN2B contained a small quantity of ceramics dating to 1200 to 700 BCE. It is likely that the canals were built contemporaneously with the dam, that is, during the early part of the Middle Preclassic period (1050 to 700 BCE).

We estimate that the total construction volume of the dam and canals is 193,000 m^3^ and the labor investment is 255,000 person-days (Supplementary Text and table S6). These numbers are an order of magnitude smaller than those for the Middle Preclassic portion of the Main Plateau: a volume of 3,630,000 m^3^ and a labor investment of 10,800,000 person-days (*18*). Although we do not know the population size of Aguada Fénix, the scale of the Main Plateau and other buildings implies that more than a thousand people participated in their construction. If a thousand people worked on the construction of the dam and canals for a few months each year, they could have completed the dam and dug the canals to their preabandonment depths within several years. Nonetheless, we should note that these estimates are based on limited excavations and auger tests, and they likely contain substantial errors. Moreover, the canal volumes and labor investment reflect their unfinished state, and up to that point, the builders had mostly dealt with soil and marl. Had they continued, they would have had to dig through hard limestone, requiring exponentially more labor.

Because the canals were left unfinished, their final intended form in terms of canal gradient is not clear. We estimate that the flow rate of Canal 2 was 1.8 m^3^/s (Supplementary Text). If the intended waterway from the eastern end of Canal 3 to the western end of Canal 5 had a constant slope, its flow rate would have been 3.9 m^3^/s. In this case, Canals 3 and 5 would not have held substantial water. More likely, the builders planned to create a more gradual slope for a large part of the waterway, comprising Canal 3 and the eastern and middle sections of Canal 5. With this design, its flow rate would have been roughly 2.0 m^3^/s, that is, a rate similar to that of Canal 2. In this case, water may have filled a substantial part of Canals 3 and 5 and then would have flowed to a steeper slope in the western part of Canal 5. However, to maintain a substantial amount of water in Canals 3 and 5, the builders would have needed to construct large embankments along the southern edge of the waterway in the areas between Canals 2 and 3 and between Canals 3 and 5 to prevent water from flowing toward the San Pedro River. We did not find any traces of such embankments in these areas.

Another issue is the small size of Laguna Naranjito. With the estimated flow rate of Canal 2, the available water held in Laguna Naranjito would have drained to the canals in 16 or 17 days, although rain and underground flow of water may have prolonged this process. Currently available evidence does not allow us to determine whether the builders intended to release lake water into the canals for a short period during rituals or whether the canals were meant to hold water for a prolonged period. In either case, as there is no evidence of large settlements, construction sites, irrigation features, or quarries in the direction of the canals, it is unlikely that the canals were built for the logistical functions of transportation or irrigation. The canals were meant to serve symbolic and ritual purposes.

### Canal deposits

After Aguada Fénix was abandoned around 700 BCE, some groups returned during the Terminal Preclassic (50 BCE to 250 CE) and the Late Classic (600 to 810 CE) periods. A substantial portion of the ceramics found in Ops. LN2A and LN2B date to the end of the Late Classic period, probably around 800 CE (figs. S8 and S9). The mound on the southern side of Canal 3 was probably built during this time. A small quantity of Terminal Preclassic ceramics was also found in Op. LN2A. It is not clear whether there is a Terminal Preclassic mound covered by the Late Classic structure. The lower layer of Op. LN2A Unit 3 contained a substantial amount of Terminal Preclassic ceramics, whereas the ratio of Late Classic ceramics increased in the upper layers. These data suggest that the lower part of the canal fills was deposited during the Terminal Preclassic period or later and that most of the sedimentation occurred during the Late Classic period or later. Elemental analysis of soil samples from the fills of Canals 3 and 5 showed high levels of phosphorus, suggesting substantial human activity in the area (Supplementary Text and table S7). However, these human inputs of phosphorus may have occurred long after the canals were abandoned.

## DISCUSSION

**Fig. 10.**
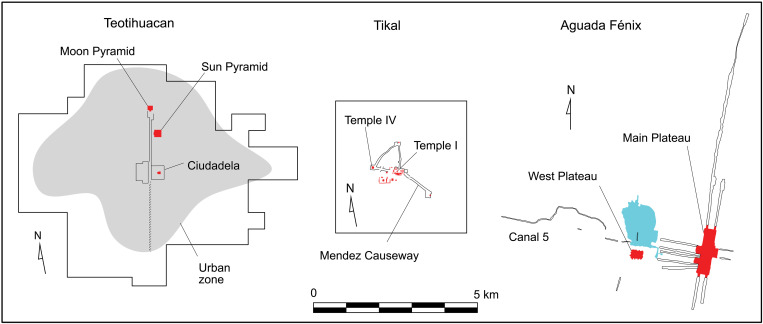
Comparison of Aguada Fénix with Teotihuacan and Tikal. The left polygon shows the area of the Teotihuacan map (*76*), and the central square indicates the Tikal map (*77*). Major construction activities at Teotihuacan took place between 200 and 550 CE (*78*, *79*) and at Tikal between 100 BCE and 810 CE (*80*), centuries after Aguada Fénix (1050 to 700 BCE).

The results of our investigation suggested that Aguada Fénix was designed as a cosmogram on the basis of north-south and east-west axes. The construction of a cosmogram representing the order of the universe and time likely motivated many people to participate in building activities without being coerced. Large construction events and collective rituals may also have involved feasting, the exchange of goods among different groups, and opportunities to meet mates, which probably provided additional incentives for people to gather. The dimension of the cosmogram, measuring 9 km by 7.5 km, is comparable to, or even greater than, those of later Mesoamerican cities (Fig. 10).

The center of this design was the E Group built in the central part of the Main Plateau, specifically marked by the cruciform cache NR10/11. Deposited in this cache, the blue, green, and yellow pigments, as well as the red and yellowish shells, represented the earliest known expression of directional color symbolism in Mesoamerica. The azurite pigment of Aguada Fénix was also the earliest known case in Mesoamerica. Prior to our study, azurite pigments had not been known before the possible example in the San Bartolo murals dating to around 100 BCE. Confirmed examples came from Classic period sites, such as Teotihuacan, Tikal, and Bonampak (*32*–*34*). Malachite pigments were found in Offering No. 12 at La Venta and possibly in the murals in Oxtotitlan Cave, both dating to the Middle Preclassic period, probably somewhat later than that of Aguada Fénix (*35*, *36*). Although yellow ochre with goethite was most likely obtained locally, there are no sources of azurite and malachite in the Maya lowlands. Malachite and azurite are known to be present at copper sources in the Mexican highlands and West Mexico (*32*, *37*). Smaller deposits of malachite and azurite are also reported from various locations in Chiapas, Tabasco, the Guatemalan highlands, Honduras, and El Salvador (*38*–*41*). Although we do not know the sources of the azurite and malachite pigments found in Cache NR11, these materials were probably obtained through trade and were highly valued. The directional color symbolism expressed in Cache NR11 of Aguada Fénix probably led to similar concepts held by the later Maya and other groups. Nonetheless, the directional colors of Aguada Fénix are different from the pattern known for the Classic to Colonial period Maya with red/east, white/north, back/west, yellow/south, and green/center (*32*, *42*, *43*). In particular, the use of green and blue pigments for different directions is intriguing as the Maya from the Classic to modern times use the term “yax” for both colors (*32*, *44*).

In addition to the directional colors, the shells placed on the western and southern sides may have symbolized water. Later Mesoamerican groups often associated the western direction with the watery underworld of the dead through which the sun entered at the end of the day. Deposits of marine organisms attributed to this concept have been found on the western side of caches and offerings, such as those at the Templo Mayor in the Aztec capital (*45*).

The builders extended the north-south and east-west axes defined by Cache NR10/11 over the landscape by marking each direction with two passageways, which may have been used for ritual processions in a way similar to ceremonial moves in and out of the center to cardinal directions practiced by the later Maya (*42*, *46*, *47*). More generally, the design of Aguada Fénix presaged the importance of processions, directional color symbolism, and the cardinal directions documented for later periods (*48*–*53*). Canals 2, 3, and 4 were possibly meant to be an extension of West Causeway 2 in a straight line. Given the apparent lack of logistical functionality, these canals most likely served symbolic purposes, involving the ritual importance of water, as seen in various examples of Mesoamerican groups (*54*–*58*).

The builders’ original plan appears to have been directing the water of the laguna raised by the dam to the west in a straight line through Canals 2, 3, and 4 and the western part of Canal 5. However, they probably realized that digging through the upland was not practical and began to dig Canal 5 by circumventing the upland. This required digging to depths of 5 to 6 m over a distance of roughly 2.5 km and to depths of 1 to 3 m over an additional 1 km or more. Given that they built large Canal 3, this might have been an achievable goal. However, Canal 5 was left unfinished, the reason for which remains unclear. This issue is complicated by the imprecision in dating, which does not allow us to determine the order of construction between the canals, the Main Plateau, and other buildings. One possibility is that the canals were built concurrently with the Main Plateau. This scenario may be supported by the radiocarbon dates of 930–820 BCE associated with the dam. The builders may have decided not to finish constructing the canals, thinking that the plan was unrealistic. After digging through soil and marl, they possibly encountered harder limestone. They may have realized that it was much harder to dig through limestone. Another possibility is that the canals were built toward the end of the Middle Preclassic occupation of Aguada Fénix, and the site was abandoned before the canals were completed. Other unfinished constructions may be present at the site, including the West Plateau, measuring 390 m by 270 m horizontally and 15 to 18 m in height, as well as a gridded area, probably made in preparation for the construction of a plaza or plateau (Fig. 1). These patterns may point to the interpretation that Aguada Fénix was abandoned when various construction projects, including Canal 5, were still in process.

These findings at Aguada Fénix offer important insights into the possibilities and limitations of human organization. On one hand, the construction of Aguada Fénix was an ambitious project, and the builders did achieve a substantial amount of work. The scale of these constructions is particularly impressive, given the dearth of substantial earlier buildings that are archaeologically recognizable in the region. Moreover, a certain portion of the builders probably retained some degree of residential mobility and may have come to Aguada Fénix seasonally from other places. Processions were most likely an important part of the rituals held at this site, but the northern corridors, which were dug into the ground and crossed wetlands in some locations, would have been unusable during the rainy seasons. We suspect that Aguada Fénix was used mainly during the dry season, possibly around 24 February, the date marked by the orientation of the E Group. These observations are consistent with the assumption that Aguada Fénix did not have prominent social inequality. Unlike Olmec sculptures that emphasized the images of rulers and supernatural beings tied to elite power, a stone sculpture and jade objects found at Aguada Fénix showed naturalistic representations of animals and a woman, which were images rooted in everyday experiences of people (*18*). Thus, Aguada Fénix adds to the observations that large-scale hydraulic features and monumental constructions could be built by groups without prominent social inequality, as indicated by the Purrón dam in Puebla and large-scale features for fishery in Belize and Amazonia (*59*–*61*).

On the other hand, the Middle Preclassic occupation of Aguada Fénix was relatively short, lasting roughly 350 years, and the canals were never completed. The construction of Aguada Fénix was likely a series of trials and adjustments. Because there were no earlier canals and dams of substantial scale in the nearby area of southeastern Mexico and Guatemala, we assume that the builders of Aguada Fénix were inexperienced in hydraulic planning and construction. In particular, they probably did not have techniques necessary to measure vertical differences accurately across long horizontal distances, although they could lay out horizontal plans over large areas, using astronomical observations.

We do not think that Aguada Fénix had rulers comparable to those of Olmec centers, but there were most likely community leaders with a certain level of prestige and power. The large cosmogram was probably designed by these leading figures, who had specialized skills and knowledge of astronomical observations and calendrical calculations (*26*, *27*). Although other groups moved between Aguada Fénix and their settlements, these calendrical specialists likely lived at Aguada Fénix year-round to conduct astronomical observations from fixed locations. They probably did not have coercive power, but their esoteric knowledge may have earned them respect, enabling them to persuade large numbers of people to participate in constructions and rituals. These specialists may also have played a central role in acquiring precious goods, such as jade and pigments, through trade. They possibly wore some of the greenstone objects as personal ornaments before they were deposited in caches. These community leaders may have formed an emergent elite, providing a prototype for later Maya rulers. The rulers and elites of Classic period Maya centers acted as holders of calendrical and other esoteric knowledge and were viewed as the embodiment of universal orders. The seeds of such political organization and ideology likely emerged in the Middle Preclassic community of Aguada Fénix.

## MATERIALS AND METHODS

### Pedestrian survey

Before the excavation and auger tests, we walked along the entire lengths of Canals 2 to 5 and the dam to examine their constructions. Although we did not find any ceramics on the ground surface in most locations, there were no indications that they were built in modern times. We also talked to local people, who confirmed that those features were not built recently. In addition, we examined the possible prehispanic mound located on the southern edge of Canal 3, which was detected in the LiDAR image (fig. S3). We observed that it was most likely built during prehispanic times. Its construction with limestone blocks and ceramics found on the surface suggested that it was a Classic period structure.

### Excavation

Excavations followed the methods established during our investigations of Ceibal and the previous seasons at Aguada Fénix (*62*). To control the proveniences of artifacts, we use a hierarchical recording system of excavation contexts, consisting of site code-operation-suboperation-unit-level-lot. A site code consists of two letters: NR for the central part of Aguada Fénix and LN for the dam and canals. An operation refers to excavations of a mound group or a similar area, suboperation to those of individual structures or a small area, unit to a horizontal division usually of 2 m by 2 m, level to a major group of stratigraphic layers, and lot to any natural or arbitrary division within a unit and a level. Operations included Op. NR5 for the E Group Plaza, Op. LN1 for the dam, Op. LN2 for Canal 3, and Op. LN3 for Canal 5. We excavated following construction sequences or natural layers whenever possible. When we encountered a fill thicker than 30 cm, like that of the cruciform pit, we divided it into multiple lots arbitrarily. We screened all excavated soils with ¼ inch or smaller mesh. From various contexts, we collected soil samples for floatation, phytolith analysis, and soil analysis.

When we exposed a floor, we cleaned it thoroughly to examine the presence of any intrusions. We excavated intrusions before we dug the floor. During the 2022 season, we noticed the western part of the cruciform pit cut into Floor 10a. During the 2023 season, we decided to expand the excavation unit to expose the entire cruciform pit.

### Auger test

We conducted auger tests using a hand-operated bucket auger along Canal 5 to examine stratigraphy and the depths to bedrock (Fig. 4 and table S4). For every 15 to 20 cm, we collected soil samples and recorded Munsell soil colors. When we reached hard bedrock, we stopped augering. We floated soil samples to collect charcoal samples. However, the quantities of organic matter in canal sediments were extremely small, and we did not find any charcoal pieces suitable for radiocarbon dating (fig. S7).

### Radiocarbon dating

The 68 radiocarbon samples from the E Group Plaza (Op. NR5) and the dam (Op. LN1) were analyzed with accelerated mass spectrometry (AMS) at the University of Tokyo Radiocarbon Dating Laboratory (data S1). All samples were treated with the acid-alkali-acid method.

We conducted the Bayesian analysis of radiocarbon dates by using the OxCal 4.4 program and the IntCal20 calibration curve (*63*–*67*) (data S2). For studies in the Maya region, some scholars recommend mixing IntCal, primarily representing the Northern Hemisphere condition, with SHCal20 for the Southern Hemisphere. However, we still do not have sufficient data to understand atmospheric mixing in the region, and we prefer to use IntCal20 alone, which is based on higher-quality calibration data. In addition, chronologies of many Mesoamerican sites are based on IntCal, and its use facilitates chronological comparisons between different regions of Mesoamerica. Elsewhere, we have discussed the methods of Bayesian analysis in detail, and other scholars have also explained it extensively (*12*, *64*, *68*–*73*). Here, we present a brief summary. Bayesian analysis serves to refine radiocarbon dates by incorporating stratigraphic information and other archaeological data. It also estimates the beginning and end dates for an occupation phase. Moreover, through the visual representations of probability distributions and statistical measures (agreement indices and outlier models), Bayesian analysis helps identify problematic dates.

In examining radiocarbon dates from our excavations, we made separate Bayesian models for individual operations, incorporating information on stratigraphic sequences as a prior. After removing obviously problematic dates (identified as outliers in data S1), we ran both general and charcoal outlier models. General outlier models identify outliers that are earlier or older than expected dates, whereas charcoal outlier models assume that outliers are older than expected dates because of inbuilt ages (*74*, *75*). At Aguada Fénix and other Mesoamerican sites, problematic dates often result from the recycling of old construction materials and the stratigraphic redeposition of old construction fills. In those cases, carbon samples give radiocarbon dates older than the dates of their final depositions. In addition, many charcoal samples found at Aguada Fénix were small or fragmented, and some of them were probably mixed with soil. The samples mixed with soil resulted in low carbon content, which in many cases made radiocarbon dates older than expected.

In Op. NR5, we encountered numerous intrusions. Although we tried to identify all intrusions and to excavate them stratigraphically, we may not have recognized some intrusions. Those intrusions could have introduced younger charcoal. We thought that the general outlier model of Op. NR5 underestimate outliers with inbuilt ages whereas the charcoal outlier model missed a few samples that may have been introduced by intrusions. Considering the results of the general and charcoal outlier models, we manually identified outliers for the final model (identified as probable outliers in data S1), assuming that a large portion of outliers resulted from the mixing of old charcoal. We determined that three samples (TKA-26486, TKA-26581, and TKA-28019, which are younger than a substantial number of dates from upper layers) as outliers caused by intrusions, animal burrows, or roots.

For Op. LN1C, the general outlier model identified one probable outlier, TKA-28030, which had low carbon content. After removing this date, the Bayesian model indicated that seven samples fell consistently between 930 and 820 BCE.

## Supplementary Materials

### The PDF file includes:

Supplementary Text Figs. S1 to S9 Tables S1 to S7 Legends for data S1 and S3 Data S2 References
