# Supplementary material for: Landscape-wide cosmogram built by the early community of Aguada Fénix in southeastern Mesoamerica
Source: Sci Adv. 2025 Nov 5;11(45):eaea2037. doi: 10.1126/sciadv.aea2037 (PMC12588264; doi:10.1126/sciadv.aea2037)
Supplement: Supplementary file 1 — Supplementary Text Figs. S1 to S9 Tables S1 to S7 Legends for data S1 and S3 Data S2 References [file sciadv.aea2037_sm.pdf]

Science Advances
AAAS

Supplementary Materials for

Landscape-wide cosmogram built by the early community of Aguada Fénix in southeastern Mesoamerica

Takeshi Inomata *et al.*

Corresponding author: Takeshi Inomata, inomata@arizona.edu

Sci. Adv. **11**, eaea2037 (2025)
DOI: 10.1126/sciadv.aea2037

The PDF file includes:

Supplementary Text
Figs. S1 to S9
Tables S1 to S7
Legends for data S1 and S3
Data S2
References

Other Supplementary Material for this manuscript includes the following:

Data S1 and S3

Supplementary Text

Lidar

The high-resolution lidar data were obtained by the National Center for Airborne Laser Mapping (NCALM) of the University of Houston. NCALM has been collecting lidar data for various parts of Mesoamerica and other regions (*81-90*). The NCALM crew collected lidar data for 109 km^2 on May 6, 2017, for 745 km^2 on June 9-17, 2019, and for an additional 5 km^2 area along Canal 5 in May, 2023 (*18, 25*). For all campaigns, the NCALM team used an Optech Titan lidar system, which is equipped with three channels of laser at wavelengths of 1550, 1064, and 532 nm (*91, 92*). The following parameters were used: a flying height of 650 m above ground level, a pulse repetition frequency (PRF) of 150 kHz, a scan frequency of 25 Hz and a scan angle of ± 30°. This configuration produced swath widths of 750 m, which were laterally overlapped by 50 %, with a flight line spacing of 345 m. These settings yielded densities of 14.7 pulses/m^2, 18.5 returns/m^2 and 10.4 ground returns/m^2. To assess the lidar height model precision, the NCALM crew compared the lidar data against 965 kinematic GPS measurements processed with differential and dual wavelengths geodetic techniques. The results indicate that the precision of the lidar models is within ± 1.9 cm (1 standard deviation). NCALM researchers classified laser points, using TerraScan software, and created a digital elevation model (DEM or bare earth model without vegetation and modern buildings) and a digital first surface model (DSM with vegetation and buildings) at horizontal spacings of 1 m for the 2017 data and 0.5 m for the 2019 and 2023 data. To examine archaeological features, we analyzed the lidar data using ArcGIS Pro 3.5.1. We applied various visualization techniques, including hillshades, slope gradient, Simple Local Relief models and Red Relief Image Map (RRIM) (*93-98*).

Ceramic Analysis

The ceramics of Aguada Fénix were similar to those from Ceibal, and we developed the ceramic typology for the site building on the one for Ceibal, which was originally established by Sabloff and was later refined by Inomata (*10, 12, 18, 99, 100*). We used Ceibal type names, such as Abelino Red, Hueche White, and Crisanto Black for ceramics that exhibited close similarities to those of Ceibal. We created new types for ceramics unique to Aguada Fénix. They include the Tiradero Group, which is characterized by thin buff to white pastes with volcanic ash temper. Some Tiradero vessels have red paint. Only a very small portion of the ceramics appear to have some affinities with materials from the Gulf Coast or Chiapas. We gave them temporary descriptive names, such as Gray volcanic ash and Gray sand. We also conducted modal analysis, particularly focusing on vessel forms, to correlate the occupation of Aguada Fénix with ceramic phases of Ceibal and other lowland Maya sites.

Pigment Analysis

Small pieces of pigments that were separated from the lump of the pigment during excavation were collected for the analysis of their composition and processing techniques. Elemental composition data was obtained through micro-X-ray fluorescence (μ-XRF) using an iXRF ATLAS M instrument. Molecular and crystalline structures of each sample were determined using a Renishaw inVia confocal Raman microscope equipped with 514 nm and 785 nm excitation lasers. Additionally, mounted dispersions of each sample were analyzed by polarized light microscopy using an Olympus BX-51 microscope to assess diagnostic optical properties (e.g., particle size and morphology, refractive indices, birefringence, etc.). Particle size distributions were quantified through Feret’s diameter measurements in ImageJ (table S1).

The following pigments from Cache NR11 were analyzed: NR5B-S1 (Lot NR5B8-5-32, yellow pigment found on the southern side); NR5B-S2 (Lot NR5B8-5-32, green pigment on the eastern side); NR5B-S3 (Lot NR5B8-5-32, blue pigment on the northern side); and NR5B-S10 (Lot NR5B8-5-29, reddish sand covering the pigments and the shells).

NR5B-S1 is a calcite-rich yellow ochre (α-FeOOH) containing fine-grained aggregates of yellow goethite (FeO(OH)), quartz, and clay particles, suggesting it was obtained from calcareous sedimentary deposits, or mixed with calcareous sediments. NR5B-S2 is a malachite pigment ($Cu_2CO_3(OH)_2$) with characteristic pale green, fine acicular particles, morphologies most commonly seen in malachite processed from secondary copper deposits. NR5B-S3 is an azurite pigment ($Cu_3(OH)_2(CO_3)_2$) with angular crystals consistent with processing by crushing and grinding. NR5B-S10 is a red ochre predominantly comprised of very fine particles of iron oxide coating quartz grains and clay minerals.

Particle size distributions were used to assess processing techniques, as particle size and shape affect color, opacity, tinting strength of paints (table S1). Fine particles (1-20 µm) and 20-50 µm (medium-grained) ranges, are often higher quality pigments, which tend to have greater vibrancy and workability compared to coarser variants (>50 µm). Measurements of Feret's diameter were derived from ImageJ analysis of photomicrographs of each sample (table S1). The yellow ochre (NR5B-S1) has the smallest and most uniformly sized particles while the red ochre (NR5B-S10) showed the most heterogeneity, consistent with the original assumption that it was an unprocessed mixture of ochre and sand taken from alluvial deposits of the Usumacinta River. In contrast, the copper-based pigments are notable for their heterogeneous particle size distributions. Fine-grained particles of azurite and malachite produce paler colored pigments, whereas medium- and coarse-grained particles are much darker in color (*101-103*). NR5B-S2 and NR5B-S3 exhibit a distinct mixture of paler fine-grained and darker and brighter medium-grained particles, suggesting attempts to optimize both color intensity and workability.

Shell Analysis

Three distinct marine shell species were included in the cruciform cache NR11 (Fig. 5). The west direction included a milk conch (*Macrostrombus costatus*) and an eroded valve of a spiny oyster (*Spondylus* sp.). The south side of the cache contained a fragmented right-side valve of a pearl oyster (*Pinctada* sp.). The milk conch and the *Spondylus* valve still maintained their red coloration and may have been brighter in the past. The red color was likely intended to distinguish the west point of the cruciform from the other colored directions. The pearl oyster was located with the yellow ochre pigment. Although the fragmented shell now has a chalky white consistency after having been buried in the ground for so long, the nacre of this genus can have a faint golden cast, which may have been part of the reason it was included with the ochre.

The three shells exhibited evidence of modifications, indicating they may have been used prior to deposition. Someone had removed the exterior lip or "wing" of the milk conch, as the edge showed evidence of carving and smoothing. It also had several light incisions or "false cuts" parallel to the final cut edge, possibly to help indicate where the final cut would be made. The wing was not present in the deposit. Similarly, the *Spondylus* valve had been cut along all edges to form a square or diamond shape. It had also been drilled to form at least two 2-mm diameter holes from the inside outward (notably, the valve had many natural holes as well from weathering and possibly marine predators, but these lacked the intentionally smoothed edges of the drilled holes). The holes indicate the shell may have been worn as an ornament. The exterior

side of the pearl oyster valve appeared to have been lightly soothed, but it was otherwise unmodified.

Phytolith Analysis

Soil samples for phytolith analysis were processed in the LEEP laboratory at the School of Anthropology, University of Arizona, following the established standard protocol. The procedure involved acid treatment with 10% hydrochloric acid (HCl), deflocculating with 5% calgon solution (sodium hexametaphosphate), removing organics with 30% hydrogen peroxide (H_2O_2), and heavy liquid separation (sodium polytungstate (SPT), density 2.35 g/mL). The extracted sample was mounted with glycerol (50/50 vol) and observed with an Olympus BX-51 microscope.

Among a total of 18 soil samples processed for phytolith analysis to date, eight were collected from Op. NR5B (the E Group Plaza), and three from Op. LN1 (the dam) (table S2, data S3). For those 11 samples, a total of 873 phytoliths were recovered, including Poaceae grasses and woody species (fig. S1). The quantities of phytoliths are smaller than those at Pajonal, a site located on the alluvial plain of the Usumacinta River that we investigated. This pattern probably reflects the poor preservation of phytoliths at Aguada Fénix due to the alkaline soils of the region characterized by limestone bedrock. Two damaged but still recognizable wavy-top rondels were observed in Op. LN1A, suggesting the presence of maize. Additionally, diagnostic spherical echinate phytoliths indicating Arecaceae (palm) was present in a notable concentration in Sample NR5B-T1 (Lot NR5B4-5-1), indicating that parts of palm were included in the fill of Floor 6 or 7. It is worth mentioning that more than half (61.7%) of the recorded phytoliths are epidermal cells. Although they are not diagnostic of specific plant species, their high proportion in the samples suggests a forest-dominated environment, since grasses typically produce fewer of these phytoliths (*104*). Their frequencies are particularly high in the samples from Cache NR11(NR5B-T28, T31, T33, and T34), which may have resulted from the use of forest resources. Additionally, opaque perforated platelets representing Asteraceae were also identified.

Some sponge spicules (n=7), which derived from aquatic habitats, were observed in samples from Op. LN1A. Their presence indicates that the clay fill of the dam was taken from the sediments in the lake or nearby wetlands. Another interesting observation is the concentration of square-based bipyramidal shaped particles that concentrated in two contexts: NR5B-T1 (n=22, Lot NR5B4-5-1) and NR5B-T31 (n=71, Lot NR5B8-5-28, dark soils under the shell in Cache NR11). They are probably calcium oxalate crystals. Calcium oxalate can be generated from plant and non-plant sources (*105*). They have multiple types of forms that are discussed as indicators in plant biology (*106*), but their relationships to specific kinds of plant exploitation and processing need further investigations.

Fossil Pollen Extraction from Soils of Canals 3 and 5 and the Wetland

We extracted fossil pollen grains from the sediment samples taken from Canals 3 and 5 and a wetland located near Canal 5, using the combined approach of physicochemical pre-treatment and subsequent cell sorting at Ritsumeikan University, Japan (*30, 31*). Our primary objective was to radiocarbon-date extracted pollen. Although we did not obtain sufficient pollen for radiocarbon dating, the results may still reflect the depositional processes of the canals.

After grinding the samples, we added 7% HCl and left them to stand at 25 °C for five days to remove the abundant carbonates contained in the samples. The samples were sieved with a 1 mm mesh to remove coarse particles and then were divided into approximately 50 g portions

to ensure efficient recovery of fossil pollen grains. After removing residual HCl, we treated the samples with 10% KOH at 90 °C for 30 minutes to eliminate organic matter. The samples were then fractionated using a heavy liquid (density: 1.78 g/ml) and a 50 μm mesh. The fine and light fraction was further treated with 10% KOH at 90 °C for 20 minutes and was sieved with a 10 μm mesh to extract only the 10–50 μm fraction.

Next, we isolated fossil pollen grains from the pre-treated samples using a SH800Z cell sorter (Sony, Tokyo, Japan) at Ritsumeikan University. All particles in the samples were excited by a coupled laser beam of 405 nm and 488 nm, and the fluorescence intensities were observed at channels of 450 ± 25 nm, 525 ± 25 nm, 600 ± 30 nm, 665 ± 15 nm, 720 ± 30 nm, and 785 ± 30 nm, as well as forward scatter (FSC) and backscatter (SSC). We identified fossil pollen grains based on these parameters and then extracted them (fig. S7). The extracted fossil pollen grains were microscopically confirmed to be of sufficient purity.

We extracted the fraction containing fossil pollen grains from samples LN2B-S3 (Canal 3 bottom), LN3A-S72 (Canal 5 bottom), and LN3B-S27 (the bottom of the wetland located north of Canal 5)(table S5). Microscopic observations showed that all of the pre-treated samples were predominantly composed of clay-sized particles, with minimal quantities of clastic materials or organic matter (fig. S7). In particular, Sample LN2B-S3 was dominated by clay-sized particles and lacked the target fraction almost entirely. In contrast, Samples LN3A-S72 and LN3B-S27 contained small quantities of particles with a diameter in the range of 10–50 μm. Sample LN3A-S72 contained a small quantity of clastic materials, but very few pollen fossils. The near absence of plant fragments also suggests that the input of organic matter into the sediments was limited.

Cell sorter observation indicated that Sample LN3B-S27 contained a greater number of particles exhibiting stronger forward scatter than Sample LN3A-S72, suggesting a higher proportion of particles larger than 10 μm. Although these coarse particles in Sample LN3B-S27 were mainly composed of low-fluorescence mineral grains, they also contained small amounts of plant fragments and fossil pollen with higher fluorescence intensity. Therefore, we extracted fossil pollen grains from the pre-treated Samples LN3A-S72 and LN3B-S27. As a result, fewer than 1,000 fossil pollen grains were recovered from each sample. These quantities of pollen grains were insufficient for accurate radiocarbon dating, and we did not conduct radiocarbon measurements. Given the small quantities of the fossil pollen grains in these samples, the depositional processes of these features require careful consideration.

Dam and Canals Volume and Labor Estimates

We calculated the fill volume of the dam and the volumes of excavated materials for the canals, following the methods used in our previous studies (*13, 18*). First, we calculated the average cross-section areas of the dam and canals, using Microstation 2024 (version 24.00.02.62). Then, we calculated the volume by multiplying the cross-section area by the length of each feature listed in table S6. We divided Canal 5 into six sections. Note that these lengths do not include the ends of features, where cross-section areas are small. Thus, they may be different from the lengths described in the main text. We also verified the fill volume of the dam by analyzing lidar data with the Polygon Volume tool of ArcGIS Pro 3.5.1.

To estimate the labor required to dig canals, we divided the cross-sections into soil and marl layers. We assumed that the soil layers were 40 cm thick in most areas, but for Canal 5 Sections 2 and 4 along the natural graben, we assumed that the soil layers were 65 cm thick. For Canal 5 Section 6, where a thick accumulation of soil was found, we used a soil thickness of 100 cm. Our estimates of labor investment followed the data obtained from experimental studies

conducted by various scholars (*107-111*). We estimated that one person could dig 2.6 m^3 of soil and 1.0 m^3 of marl a day. We estimated that one person could transport 0.14 m^3 of stones over a mean distance of 500 m for the construction of the dam. In constructing the canals, the builders piled the excavated materials along the edges of the canals. We assumed that one person could carry 5.1 m^3 of soil and 3.3 m^3 of marl over an average distance of 20 m a day.

We should note that these estimates are based on limited excavations and auger tests, and there may be substantial errors. As the builders dug deeper, they went through soil, soft marl, harder marl, and very hard limestone. Our calculations did not account for the effort required to dig through limestone, and they may substantially underestimate labor investment where the builders may have encountered limestone, particularly in Canals 2 and 3.

<u>Canal</u> <u>Flow</u> <u>Rate</u>

The flow of water through a canal can be estimated with the Manning's formula.

$$V = \frac{1}{n} R^{\frac{2}{3}} S^{\frac{1}{2}}$$

Where
V = velocity (m/s)
n = Manning's roughness coefficient
R = hydraulic radius = $\frac{A}{P}$ (where A = cross-sectional area; P = wetted perimeter)
S = stream slope

$$Q = AV$$

Where
Q = flow rate (m^3/s)

To make a rough estimate of the flow rate of Canal 2, we assume that its narrowest section, where the canal cuts through the dam, largely determines the flow rate. Then, we use the following values:
n = 0.04
A = 2.5
P = 5.4
S = 0.0023

$$V \approx 0.72 \text{ (m/s)}; Q \approx 1.8 \text{ (m}^3\text{/s)}$$

If the final waterway had had a constant slope from the eastern end of Canal 3 to the western end of Canal 5, we may assume:
n = 0.04
A = 7.5
P = 15.3
S = 0.0011

$$V \approx 0.52 \text{ (m/s)}; Q \approx 3.9 \text{ (m}^3\text{/s)}$$

In this case, the flow rate of Canal 2 would have been substantially smaller than that of Canals 3 and 5.

More likely, the builders intended to make a gentle slope for the eastern and middle parts of Canal 5. Then, we may assume:

$n = 0.04$

$A = 7.5$

$P = 15.3$

$S = 0.00029$

$$V \approx 0.26 \text{ (m/s)}; Q \approx 2.0 \text{ (m}^3\text{/s)}$$

In this case, the flow rate of the waterway connecting Canal 3 and the eastern and middle sections of Canal 5 would have been similar to that of Canal 2.

We can also estimate the volume of water in Laguna Naranjito raised by the dam that could flow to the canal (water above the elevation of the Canal 2 bottom where it crossed the dam) roughly at 2,600,000 m^3. At the flow rate of Canal 2 calculated above, this amount of water could have been discharged in 16 to 17 days. Although rain and surface and underground flows of water would have supplied additional water to the lake, it is unlikely that the canals would have held water constantly at this flow rate.

<u>Elemental Analysis of Soil from Canal 5 and the Wetland</u>

We conducted X-ray fluorescence (XRF) in the Soils & Geoarchaeology laboratory at the University of Texas at Austin. Soil samples were initially air-dried, then oven-dried at 32 °C, an average ambient temperature for the study site, to remove excess moisture. Rock and root fragments were removed, and soil aggregates were ground and sieved to 250 microns. Samples were homogenized prior to subsampling for each respective test. The inorganic geochemistry of the samples was measured with XRF, using an Olympus Benchtop BTX Profiler in both Soils and Geochemistry modes, and we report these in Soils mode (table S7). We packaged ~ 10g of sample into each plastic container with a transparent film cover. X-ray beams were directed at each sample, exciting and ejecting an inner shell electron, which was replaced by an outer shell electron, emitting a distinctive fluorescent signal that was measured for each element. We analyzed for 35 separate elements but focused on phosphorous because of its strong connection to human activity (*112*).

The range of soil phosphorus in the samples from Auger Tests LN3B1 and LN3B2 (Canal 5) was 3570 to 6909 ppm with a mean of 5056, which increased with depth (table S7). At Auger Test LN3B3 (wetland), soil phosphorous ranged from 4089 to 7218 ppm with a mean of 5662, peaking at the surface and at 3.5 m. At Auger Test LN3B9 (Canal 5), soil phosphorus ranged from 4669 to 7600 ppm with a mean of 6178, peaking at 85 cm and declining below 215 cm in the profile. The crustal mean in these contexts is 700 ppm, and soil parent materials (background levels) account for 42% of variance. In one key study, tropical soils ranged mostly below 1000 ppm, except on alkali basalts as in Hawaii (*113*). Thus, the phosphorous content of soils of these canals appears to be up to an order of magnitude higher, which is indicative of human inputs throughout these soil sequences (*112*). It should be noted that a large part of the canal fills was deposited probably centuries or millennia after the abandonment of the canals as indicated by the ceramic data.

Provenance

1) All artifacts and ecofacts were collected at the stie of Aguada Fénix, Municipality of Balancán, State of Tabasco, Mexico, during our fieldwork between May 2017 and June 2025.

2) All artifacts and ecofacts were collected from archaeological deposits through our scientific excavations, which indicates that they are authentic. Our subsequent ceramic analysis, paleobotanical analysis, faunal analysis, and soil analysis confirm their authenticity. We determined their age through ceramic dating and radiocarbon dating.

3) Following the Mexican laws, we will submit all artifacts to the Instituto Nacional de Antropología e Historia (INAH). Those who wish to access them should contact INAH.

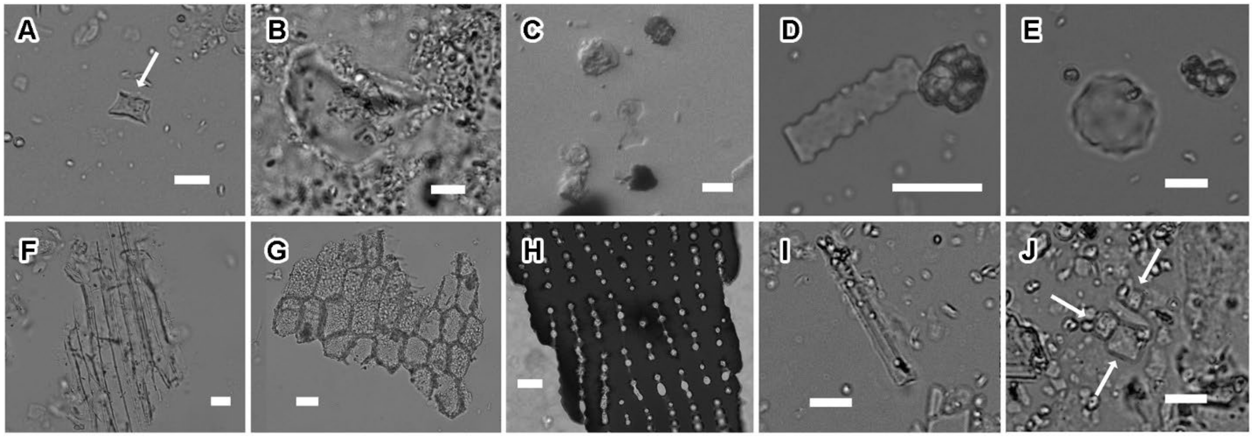

Fig. S1.

Examples of phytolith morphotype. Scale: **(D, F-H)** 20µm, others 10µm. **(A)** wavy-top rondel. **(B)** common bulliform. **(C)** bilobate. **(D)** elongate. **(E)** Spheroid echinate. **(F, G)** epidermal cells. **(H)** opaque perforated platelets. **(I)** sponge spicule. **(J)** Square-based bipyramidal crystals.

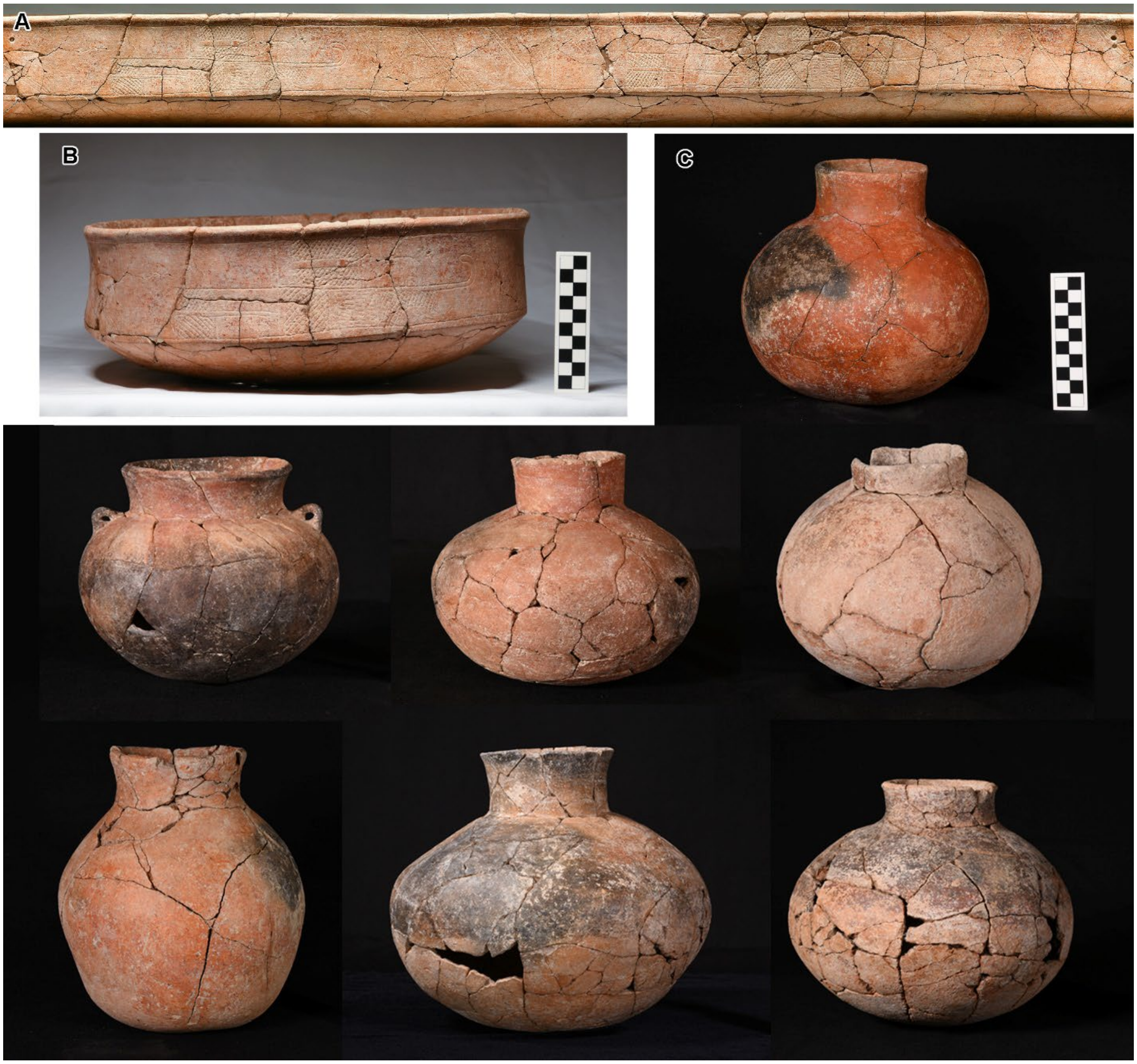

Fig. S2.

Ceramic vessels found in Cache NR4. The cache contained one dish and ten jars, three of which have not yet been restored due to poor preservation. **(A)** Rollout view of the incised motifs on the dish. **(B)** Dish with incised motifs, found in the center of the cache. **(C)** Jars found in the cache. Note that they exhibit different forms and production techniques, suggesting that they were made by different groups or individuals.

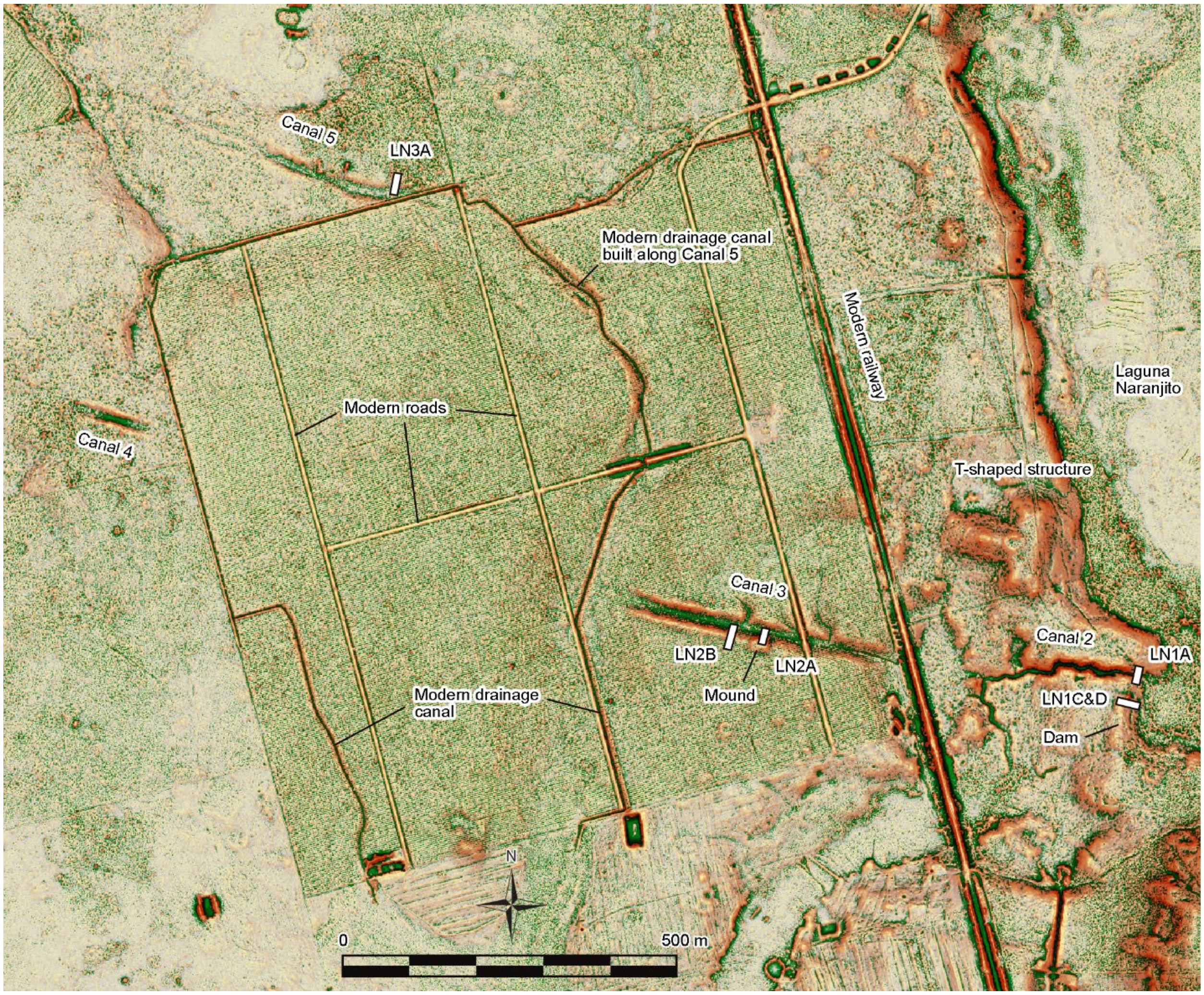

Fig. S3.

Lidar image of the dam and canals. It is shown in Red Relief Image Map (RRIM) visualization for enhanced visibility (38). Locations of excavations are shown in white.

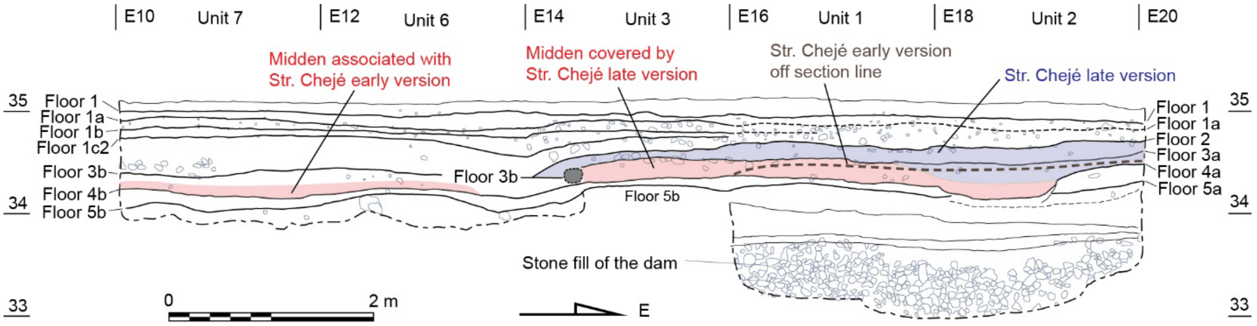

Fig. S4.

North profile of Op. LN1C in the central part of the dam. It shows the stratigraphic relationships between the fill of the dam, middens, and floors.

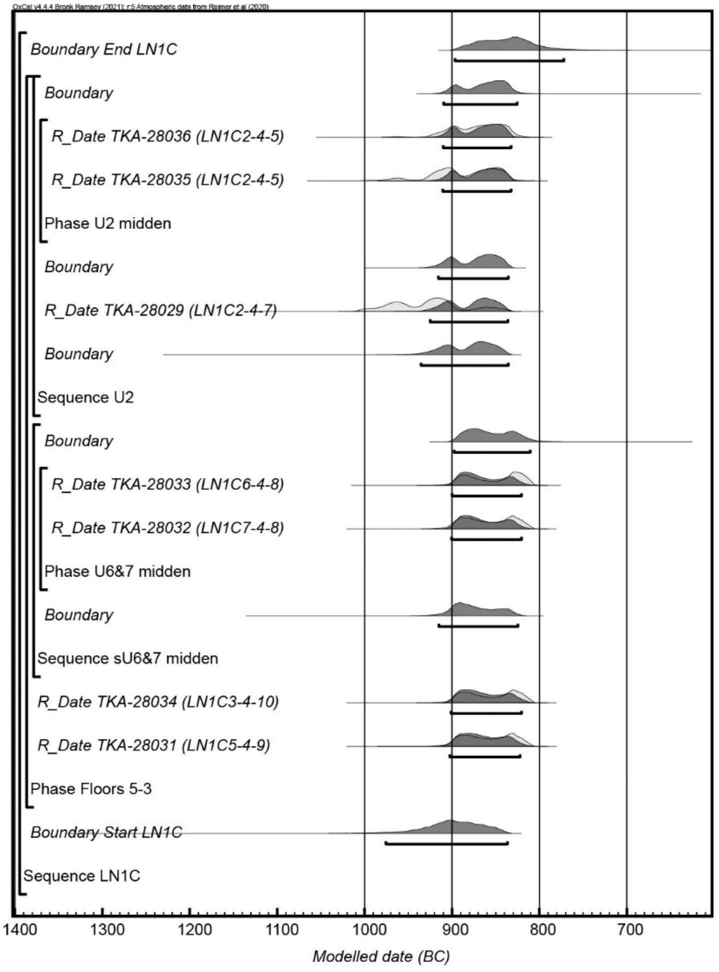

Fig. S5.

Bayesian modeled radiocarbon dates from Op. LN1C. Outliers have been removed. (see data S1 and S2).

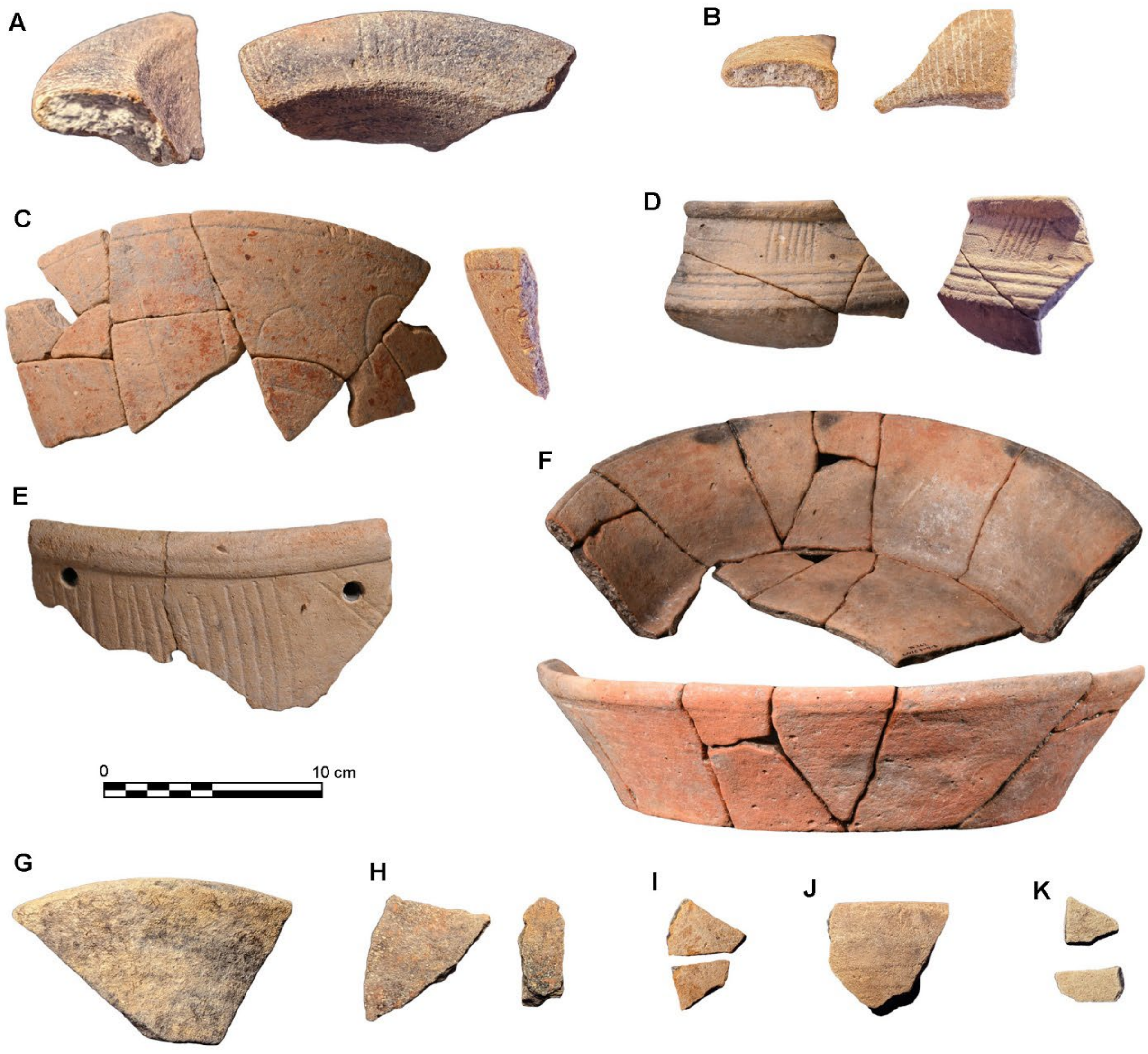

Fig. S6.

Ceramics from the excavation of the dam (Op. LN1) and the canals (Ops. LN2 and LN3) dating to 1050-700 BC. (A-F) Ceramics found in the middens above the dam (Op. LN1C). **(A)** Pico de Oro Incised with post-slip incisions and a horizontally everted rim. **(B)** Comistun Incised with post-slip incisions and a horizontally everted rim. **(C)** Pico de Oro Incised with post-slip incisions. **(D)** Comistun Incised with pre-slip incisions. **(E)** Comistun Incised with post-slip incisions. **(F)** Pico de Oro Incised. **(G-J)** Ceramics found at the bottom of Canal 2 (Op. LN2B). **(G)** Río Pasión ware with characteristic black paste (LN2B2). **(H)** Abelino Red (LN2B2). **(I)** Huetche White (LN2B3). **(J)** Río Pasión ware with characteristic black paste (LN2B4). **(K)** Ceramics found at the bottom of Canal 3 (LN3A1): Río Pasión ware with characteristic black paste.

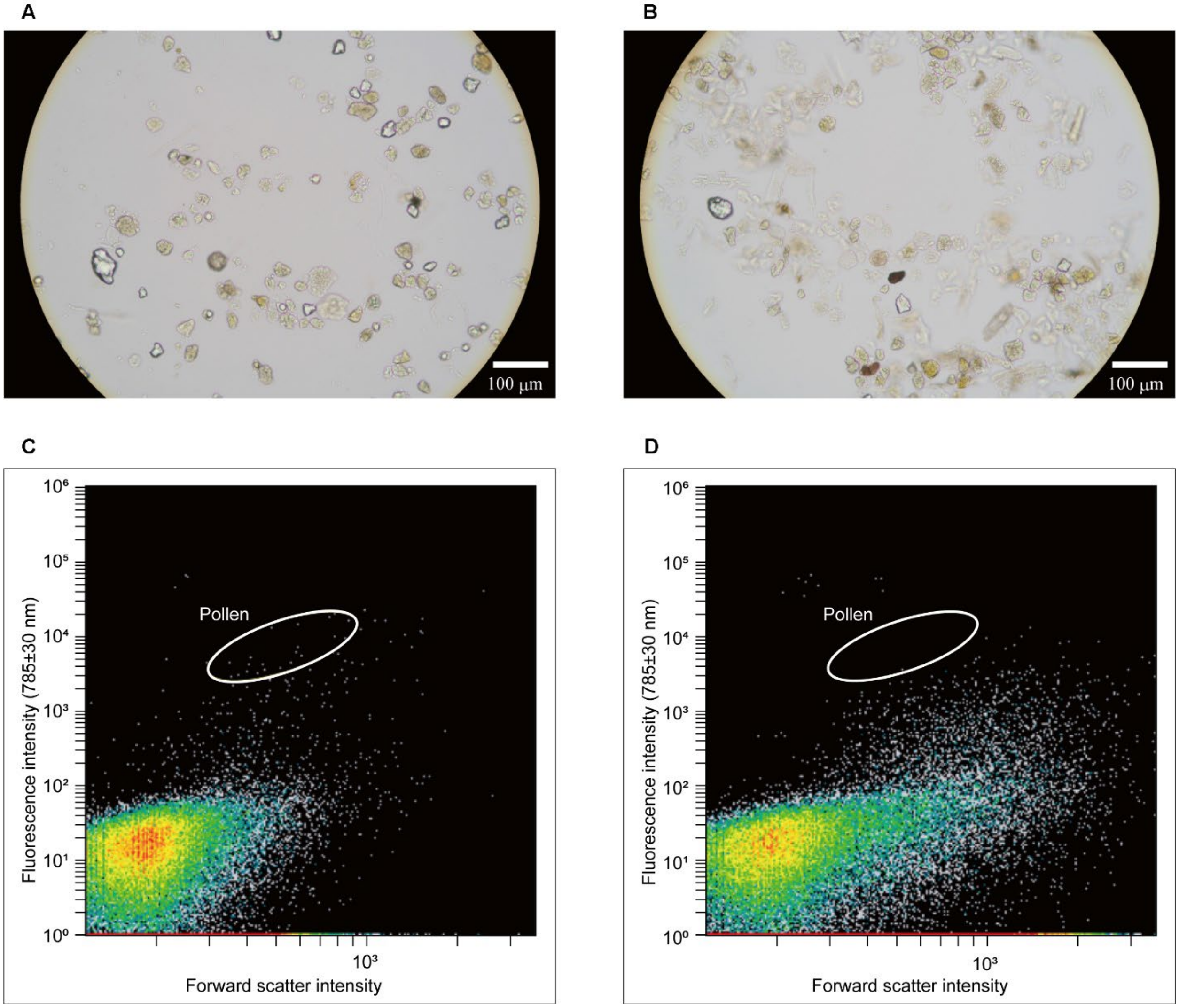

Fig. S7.

Microscopic images and scatterplots of particles in soil samples from Canal 5 and the wetland. **(A)** Microscopic image of Sample LN3A-S72 (bottom of Canal 5). **(B)** Microscopic image of Sample LN3B-S27 (bottom of the wetland). **(C&D)** Distribution of fluorescence versus forward scatter intensity. **(C)** Sample LN3A-S72. **(D)** Sample LN3B-S27. Each plot shows 100,000 particles, where dots represent individual particles and color reflects relative density. The ellipse gates show the region where fossil pollen grains are generally expected (*31*). Microscopic images and scatterplots both indicate that fossil pollen grains are largely absent.

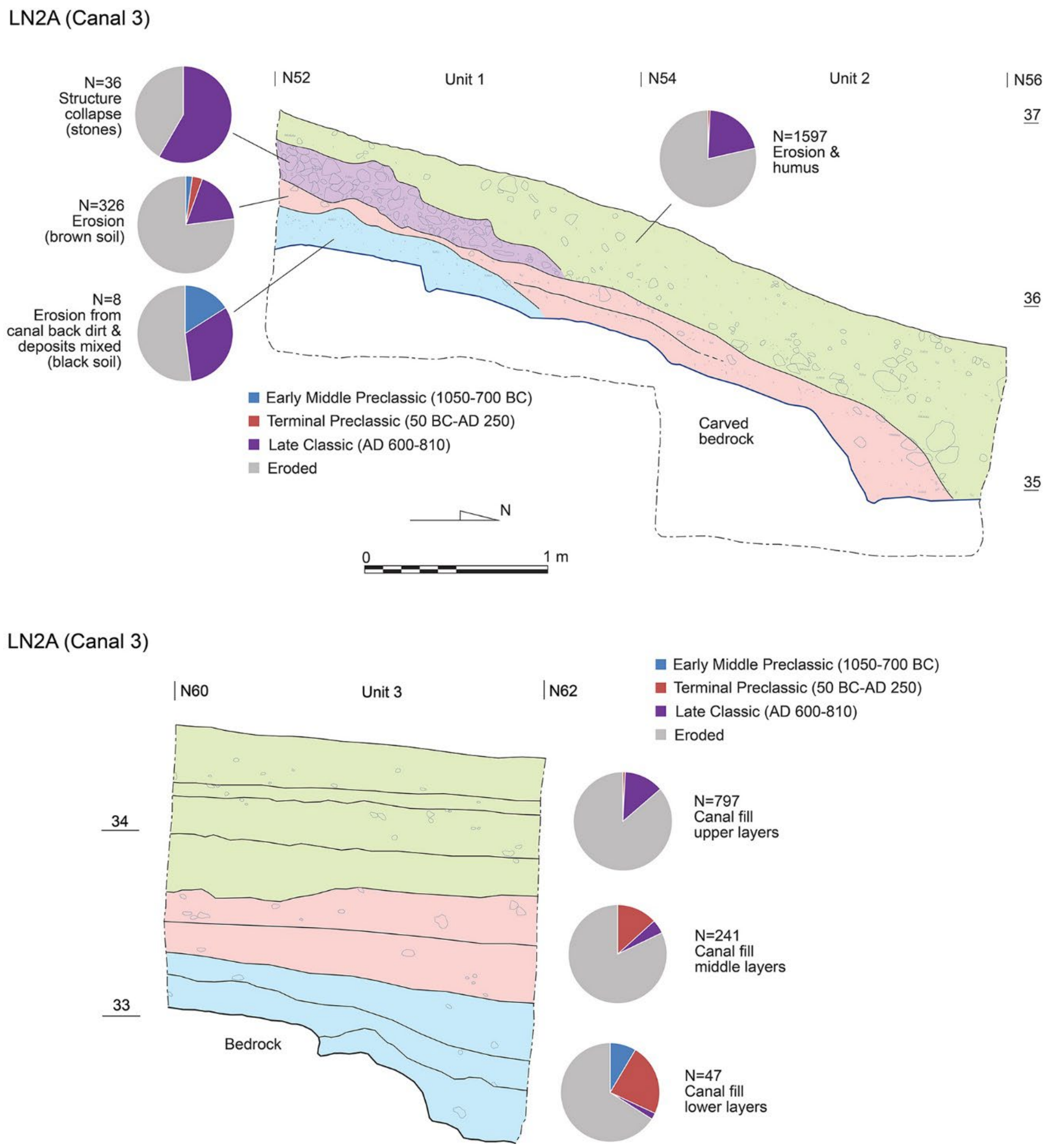

Fig. S8.

Ceramics found in Op. LN2A (Canal 3) by period and stratigraphy. Units 1 and 2 contain layers resulting from the erosion of the canal back dirt and that of the structure built on the southern bank of the canal. The collapse layer of the structure mostly contains Late Classic ceramics, with the majority dating to around AD 800, which likely represents the date of its occupation. In Unit 3, placed on the slope toward the center line of the canal, we found a mixture of early Middle Preclassic and Terminal Preclassic ceramics in the lower layers and Late Classic pottery in the upper layers. The lower part of the canal fill was likely deposited during the Terminal Preclassic period or later, and the upper layers formed during the Late Classic period or later.

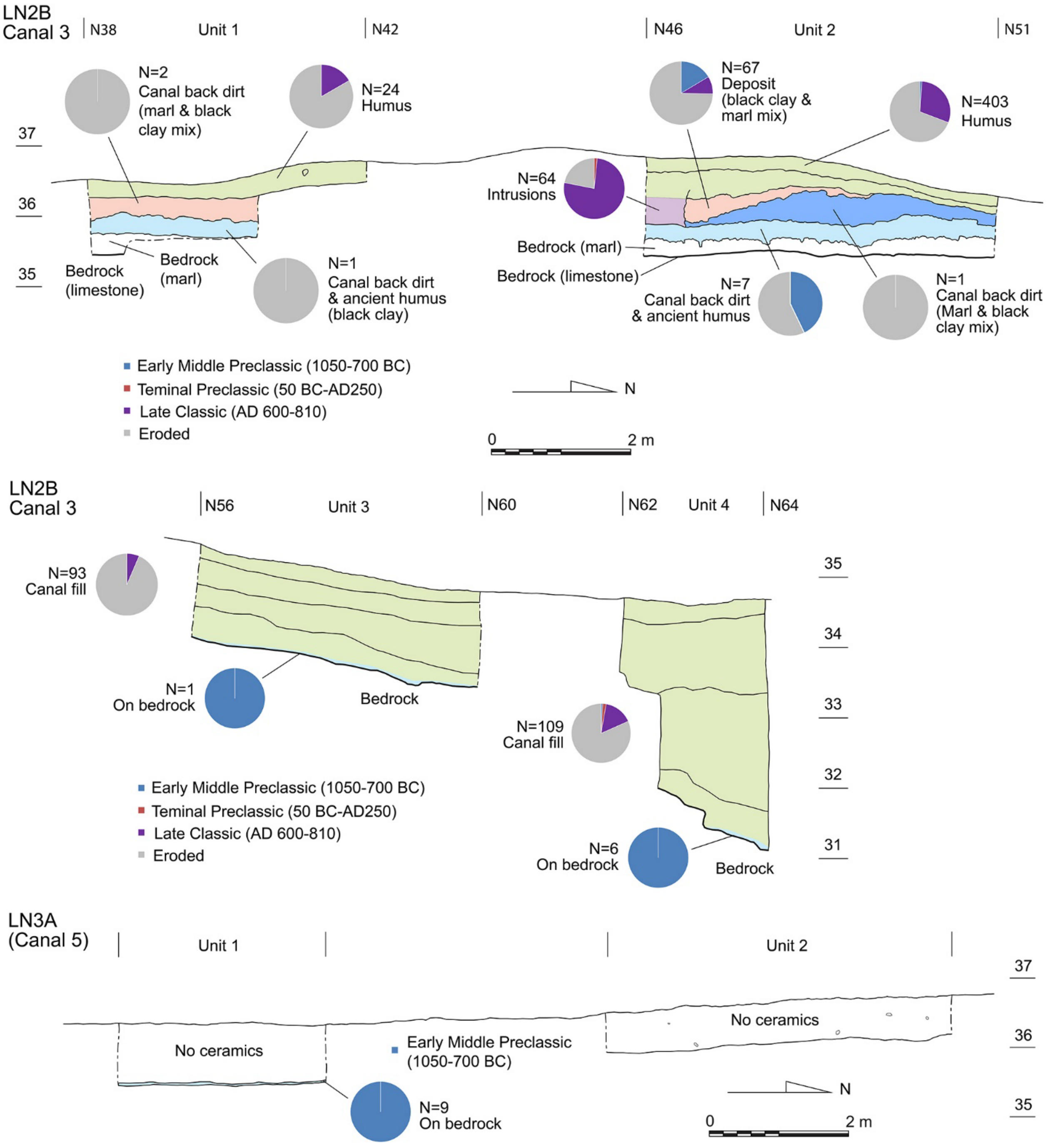

Fig. S9.

Ceramics found in Ops. LN2B (Canal 3) and LN3A (Canal 5). The canal back dirt excavated in LN2B Units 1 and 2 contained a small quantity of ceramics, with diagnostic ones all dating to the early Middle Preclassic period. We also found a small number of early Middle Preclassic ceramics at the bottom of Canals 3 and 5. These findings indicate that the canals were constructed during the early Middle Preclassic period, contemporaneous with the dam and the Main Plateau.

Table S1.
Particle size distributions of pigments using Feret's diameter.

Sample #	# of Particles Measured	Min	Max	Mean	SD
NR5B-S1 (yellow pigment)	2877	1 µm	24 µm	3.288 µm	0.359 µm
NR5B-S2 (green pigment)	634	1 µm	31 µm	5.34 µm	4.722 µm
NR5B-S3 (blue pigment)	424	1 µm	43 µm	5.296 µm	5.835 µm
NR5B-S10 (red sand)	758	1 µm	59 µm	5.921 µm	6.614 µm

Table S2.
Soil samples for phytolith analysis.

Sample	Lot	Context
LN1A-T2	LN1A1-4-2	White layer under the clay-stone fill of the dam
LN1A-T3	LN1A1-4-3	Black soil layer below the white layer
LN1C-T1	LN1C7-4-10	Midden associated with a structure on the dam
NR5B-T1	NR5B4-5-1	Floor 6 or 7 fill
NR5B-T2	NR5B4-5-4	Floor 9 fill
NR5B-T5	NR5B3-6-9	Floor 18 surface and matrix
NR5B-T13	NR5B3-7-8	Black soil on bedrock below Floor 22
NR5B-T28	NR5B8-5-29	Red sand in the southern part of Cache NR11
NR5B-T31	NR5B8-5-28	Dark soil under the shell in the western part of Cache NR11
NR5B-T33	NR5B7-5-16	Red sand in the northern part of Cache NR11
NR5B-T34	NR5B8-5-30	Red sand in the eastern part of Cache NR11

Table S3.
Radiocarbon Dates on charcoal samples from Laguna Naranjito at Core 2. (see Fig. 7).

Lab Num	Core depth (m)	pMC	Uncalibrated radicarbon date (bp)	Calibrated date range (95.4 %)
TKA-22506	2.05-2.30	108.332 ± 0.283	-642±20	AD 2001 - 2002
TKA-22507	2.30-2.52	105.168 ± 0.300	-404±22	AD 1956 - 1957, 2007 - 2009
TKA-22484	2.52-2.60	101.202 ± 0.344	-88±27	AD 1955 - 1960
TKA-22731	3.4	164.657 ± 0.565	-4006±27	AD 1963, 1966 - 1967

Table S4.
Auger tests of Canal 5 and the wetland.

Auger point	Context	Current ground elevation (m)	Depth to bedrock (m)
LN3B1	Outside Canal 5, north of LN3A	36.97	0.55
LN3B2	Inside Canal 5	36.18	1.16
LN3B3	Wetland north of Canal 5	35.79	4.00
LN3B4	Inside Canal 5	35.10	1.10
LN3B5	Outside Canal 5, north of LN3B4	35.85	0.56
LN3B6	Inside Canal 5	36.82	0.63
LN3B7	Outside Canal 5, south of LN3B6	37.39	0.13
LN3B8	Outside Canal 5, south of LN3B6	37.43	0.30
LN3B9	Inside Canal 5	29.61	3.09
LN3B10	Outside Canal 5, south of LN3B9	29.46	0.80

Table S5.
Fossil pollen extraction from soils of Canals 3 and 5 and the wetland.

Sample	Lot	Context	Depth (m)	Weight (wet g)	Pollen frequency
LN2B-S3	LN2B4-1-1	Canal 3 bottom	3.20	100.3	N/A
LN3A-S72	LN3A1-1-3	Canal 5 bottom	0.72	99.7	<1000
LN3B-S27	LN3B3-21-1	Wetland bottom	3.08	101.5	<1000

Table S6.
Volume and labor estimates for the dam and canals.

Feature	Operation		Length (m)	Soil	Marl	Total
Dam	LN1A, LN1C		120			
		Volume (m^3)			7,200	
		Digging (person-day)			7,200	
		Carrying (person-day)			51,429	
		Total labor (person-day)			58,629	58,629
Canal 2	LN1A		280			
		Volume (m^3)		1,736	9,800	
		Digging (person-day)		668	9,800	
		Carrying (person-day)		340	2,970	
		Total labor (person-day)		1,008	12,770	13,778
Canal 3	LN2A, LN2B		390			
		Volume (m^3)		3,354	22,230	
		Digging (person-day)		1,290	22,230	
		Carrying (person-day)		658	6,736	
		Total labor (person-day)		1,948	28,966	30,914
Canal 4			120			
		Volume (m^3)		840	4,188	
		Digging (person-day)		323	4,188	
		Carrying (person-day)		165	1,269	
		Total labor (person-day)		488	5,457	5,945
Canal 5 Section 1	LN3A, LN3B1		1,180			
		Volume (m^3)		11,446	18,054	
		Digging (person-day)		4,402	18,054	
		Carrying (person-day)		2,244	5,471	
		Total labor (person-day)		6,647	23,525	30,172
Canal 5 Section 2 (graben)			200			
		Volume (m^3)		3,120	1,620	
		Digging (person-day)		1,200	1,620	
		Carrying (person-day)		612	491	
		Total labor (person-day)		1,812	2,111	3,923
Canal 5 Section 3	LN3B4		500			
		Volume (m^3)		6,900	15,000	
		Digging (person-day)		2,654	15,000	
		Carrying (person-day)		1,353	4,545	
		Total labor (person-day)		4,007	19,545	23,552

Feature	Operation		Length (m)	Soil	Marl	Total
Canal 5 Section 4 (graben)			490			
		Volume (m^3)		7,644	3,969	
		Digging (person-day)		2,940	3,969	
		Carrying (person-day)		1,499	1,203	
		Total labor (person-day)		4,439	5,172	9,611
Canal 5 Section 5			650			
		Volume (m^3)		6,305	18,980	
		Digging (person-day)		2,425	18,980	
		Carrying (person-day)		1,236	5,752	
		Total labor (person-day)		3,661	24,732	28,393
Canal 5 Section 6	LN3B9		1,000			
		Volume (m^3)		21,000	29,200	
		Digging (person-day)		8,077	29,200	
		Carrying (person-day)		4,118	8,848	
		Total labor (person-day)		12,195	38,048	50,243
Total volume (m^3)						192,586
Total labor (person-day)						255,158

Table S7.
Phosphorus levels of soil samples from Canal 5 and the wetland.

Auger Test	Context	Sample	Lot	Depth (m)	P (ppm)
LN3B1	Canal 5	LN3B-S1	LN3B1-1-1	0.15	4902
LN3B1	Canal 5	LN3B-S2	LN3B1-2-1	0.30	4943
LN3B1	Canal 5	LN3B-S4	LN3B1-5-1	0.50	5724
LN3B2	Canal 5	LN3B-S9	LN3B2-5-1	0.71	6909
LN3B2	Canal 5	LN3B-S10	LN3B2-6-1	0.88	3570
LN3B2	Canal 5	LN3B-S11	LN3B2-7-1	1.00	4289
LN3B3	Wetland	LN3B-S13	LN3B3-1-1	0.15	7218
LN3B3	Wetland	LN3B-S15	LN3B3-3-1	0.43	5297
LN3B3	Wetland	LN3B-S17	LN3B3-5-1	0.89	6576
LN3B3	Wetland	LN3B-S19	LN3B3-9-1	1.45	6597
LN3B3	Wetland	LN3B-S21	LN3B3-12-1	1.88	5002
LN3B3	Wetland	LN3B-S23	LN3B3-14-1	2.13	5386
LN3B3	Wetland	LN3B-S25	LN3B3-17-1	2.54	5149
LN3B3	Wetland	LN3B-S26	LN3B3-19-1	2.80	4993
LN3B3	Wetland	LN3B-S28	LN3B3-23-1	3.35	6309
LN3B3	Wetland	LN3B-S30	LN3B3-27-1	3.94	4089
LN3B9	Canal 5	LN3B-S48	LN3B9-1-1	0.15	6713
LN3B9	Canal 5	LN3B-S50	LN3B9-3-1	0.54	7539
LN3B9	Canal 5	LN3B-S52	LN3B9-5-1	0.85	7600
LN3B9	Canal 5	LN3B-S54	LN3B9-7-1	1.15	6519
LN3B9	Canal 5	LN3B-S56	LN3B9-9-1	1.47	6430
LN3B9	Canal 5	LN3B-S58	LN3B9-11-1	1.70	5702
LN3B9	Canal 5	LN3B-S60	LN3B9-13-1	1.97	6398
LN3B9	Canal 5	LN3B-S62	LN3B9-15-1	2.15	5502
LN3B9	Canal 5	LN3B-S66	LN3B9-19-1	2.73	5561
LN3B9	Canal 5	LN3B-S67	LN3B9-20-1	2.85	5323
LN3B9	Canal 5	LN3B-S68	LN3B9-21-1	3.00	4669

Data S1. (separate file)
Radiocarbon dates from Op. NR5 (E Group Plaza) and Op. LN1C (dam).

Data S2. (below)
Oxcal code for the Bayesian analysis of radiocarbon dates.

Data S3. (separate file)
Results of the phytolith analysis.

Data S2. Oxcal code for the Bayesian analysis of radiocarbon dates

This section contains the following models:
NR5 Final Model
NR5 General Outlier Model
NR5 Charcoal Model
LN1C Final Model
LN1C General Outlier Model

The Bayesian models were processed with Oxcal 4.4 (https://c14.arch.ox.ac.uk/oxcal.html) (See the materials and methods, Data S1).

NR5 Final Model

```
Plot()
 {
 Sequence("NR5AB")
  {
  Boundary("Start E-Group Plaza");
  Sequence("Middle Preclassic")
   {
   Boundary("Start Floor 23");
   Phase("Floor 23 fill")
    {
    R_Date("TKA-26589 NR5B3-7-9 black clay",2834,20);
    R_Date("TKA-26590 NR5B3-7-9 black clay",2810,21);
    R_Date("TKA-20669 NR5A1-7-8 Floor 22 fill", 2834, 20);
    R_Date("TKA-26901 NR5A1-7-9 black clay",2787,20);
   };
   Boundary("Floor 23-Floor 22");
   Phase("Floor 22 fill cuadros")
    {
    R_Date("TKA-26588 NR5B1-7-7 earthen fill",2832,21);
    R_Date("TKA-26587 NR5B1-7-6 earthen fill",2814,20);
    R_Date("TKA-26586 NR5B1-7-6 earthen fill",2799,21);
    R_Date("TKA-26583 NR5B3-7-5 Floor 22 fill",2817,21);
    R_Date("TKA-20668 NR5A1-7-5 Under Floor 22", 2805, 21);
   };
   Boundary("Floor 22-Floor 21");
   Phase("Floor 20 fill")
    {
    R_Date("TKa-26484 NR5B1-7-3 Floor 20 fill",2800,21);
    R_Date("TKa-26485 NR5B1-7-3 Floor 20 fill",2795,21);
   };
   Boundary("Floor 20-Floor 19");
   Phase("Floor 19 fill Cuadros")
    {
    R_Date("TKa-26479 NR5B1-7-1 Cuadros",2785,20);
    R_Date("TKa-26478 NR5B1-6-10 Floor 19 fill",2811,21);
   };
```

```
Boundary("Floor 19-Floor 18");
R_Date("TKa-26477 NR5B3-6-9 On Floor 19",2755,20);
Boundary("Floor 18-Floor 16");
R_Date("TKa-26471 NR5B2-6-8 Floor 14 fill",2746,21);
Boundary("Floor 14-Floor 11");
R_Date("TKa-26469 NR5B2-6-2 Floor 11 fill",2784,20);
Boundary("Floor 11-Floor 10");
Phase("Cruciform&on Floor 10")
{
 R_Date("AA-114537  NR5B3-5-4  On Floor 10",2748,52);
 Sequence("Cruciform")
 {
  Boundary();
  Phase("Cruciform fill")
  {
   R_Date("TKA-28024 (NR5B8-5-6)",2714,20);
   R_Date("TKA-28025 (NR5B8-5-10)",2711,20);
   R_Date("TKA-28026 (NR5B8-5-10)",2710,20);
  };
  Boundary();
 };
};
Boundary("Floor 10-Floor 9");
Phase("Floor 9")
{
 R_Date("TKa-26482 NR5B5-5-7 Floor 9 fill",2752,21);
 R_Date("TKa-26483 NR5B6-5-2 Floor 9 fill",2729,20);
 R_Date("AA-114529  NR5B1-5-2  On Floor 9/fill",2768,26);
};
Boundary("Floor 9-Caches");
Phase("Cache NR6,7,8,9")
{
 Sequence("Floors 9-6")
 {
  Boundary("Floor 8-Trench");
  Phase("Trench-Cache NR6")
  {
   Sequence("Trench-Floor6")
   {
    R_Date("TKA-26584 NR5B5-5-10 Trench fill",2697,21);
    R_Date("AA-114530  NR5B4-4-8  On Floor 6",2499,25);
   };
   Sequence("S Cache NR6")
   {
    Boundary();
    Phase("Cache NR6")
    {
     R_Date("TKa-26473 NR5B5-5-4 Cache NR6",2742,20);
     R_Date("TKa-26476 NR5B5-5-4 Cache NR6",2705,21);
    };
    Boundary();
```

```
      };
     };
     Boundary();
    };
    Sequence("Caches 7,8,9")
    {
     Boundary();
     Phase("Caches NR7,8,9")
     {
      R_Date("TKA-28018 (NR5B8-5-3)",2528,21);
      Sequence("Cache NR8")
      {
       R_Date("TKA-28020 NR5B10-5-3 Cache NR8",2696,21);
       R_Date("TKA-28022 (NR5B10-5-6)",2672,21);
       Before()
       {
        Date("=TKA-26584 NR5B5-5-10 Trench fill");
       };
      };
     };
     Boundary();
     Before()
     {
      Date("=AA-114530  NR5B4-4-8  On Floor 6");
     };
    };
   };
   Boundary("End Floor 6");
  };
  Boundary("End Middle Preclassic NR5A");
  Boundary("Start Late-Terminal Preclassic NR5A");
  R_Date("TKA-20634 NR5A2-4-4 On Floor 4", 2055, 22);
  Boundary("End Late-Terminal Preclassic NR5A");
 };
};
```

NR5 General Outlier Model

```
Plot()
 {
 Outlier_Model("General",T(5),U(0,4),"t");
 Sequence("NR5AB")
  {
  Boundary("Start E-Group Plaza");
  Sequence("Middle Preclassic")
   {
   Boundary("Start Floor 23");
   Phase("Floor 23 fill")
    {
```

```
 R_Date("TKA-26589 NR5B3-7-9 black clay",2834,20)
 {
  Outlier("General",0.05);
 };
 R_Date("TKA-26590 NR5B3-7-9 black clay",2810,21)
 {
  Outlier("General",0.05);
 };
 R_Date("TKA-20669 NR5A1-7-8 Floor 22 fill", 2834, 20)
 {
  Outlier("General",0.05);
 };
 R_Date("TKA-26901 NR5A1-7-9 black clay",2787,20)
 {
  Outlier("General",0.05);
 };
};
Boundary("Floor 23-Floor 22");
Phase("Floor 22 fill cuadros")
{
 R_Date("TKA-26588 NR5B1-7-7 earthen fill",2832,21)
 {
  Outlier("General",0.05);
 };
 R_Date("TKA-26587 NR5B1-7-6 earthen fill",2814,20)
 {
  Outlier("General",0.05);
 };
 R_Date("TKA-26586 NR5B1-7-6 earthen fill",2799,21)
 {
  Outlier("General",0.05);
 };
 R_Date("TKA-26583 NR5B3-7-5 Floor 22 fill",2817,21)
 {
  Outlier("General",0.05);
 };
 R_Date("TKA-20668 NR5A1-7-5 Under Floor 22", 2805, 21)
 {
  Outlier("General",0.05);
 };
};
Boundary("Floor 22-Floor 21");
Phase("Floor 21 fill")
{
 R_Date("TKa-26486 NR5B3-7-4 Floor 21 fill",2760,21)
 {
  Outlier("General",0.05);
 };
 R_Date("TKA-26581 NR5B3-7-4 Floor 21 fill",2747,20)
 {
  Outlier("General",0.05);
```

```
 };
};
Boundary("Floor 21-Floor 20");
Phase("Floor 20 fill")
{
 R_Date("TKa-26484 NR5B1-7-3 Floor 20 fill",2800,21)
 {
  Outlier("General",0.05);
 };
 R_Date("TKa-26485 NR5B1-7-3 Floor 20 fill",2795,21)
 {
  Outlier("General",0.05);
 };
};
Boundary("Floor 20-Floor 19");
Phase("Floor 19 fill Cuadros")
{
 //R_Date("TKA-20638 NR5A1-6-13 Floor 19 fill", 3800, 25){Outlier();};
 R_Date("TKa-26479 NR5B1-7-1 Cuadros",2785,20)
 {
  Outlier("General",0.05);
 };
 R_Date("TKa-26478 NR5B1-6-10 Floor 19 fill",2811,21)
 {
  Outlier("General",0.05);
 };
};
Boundary("Floor 19-Floor 18");
R_Date("TKa-26477 NR5B3-6-9 On Floor 19",2755,20)
{
 Outlier("General",0.05);
};
Boundary("Floor 18-Floor 16");
Phase("Floor 18-Floor 11")
{
 Sequence("5A F14-13")
 {
  Boundary("Start Floor 14");
  R_Date("TKA-20637 NR5A1-6-6 On Floor 16", 2845, 23)
  {
   Outlier("General",0.05);
  };
  R_Date("TKA-20635 NR5A2-6-4 On Floor 14", 2835, 25)
  {
   Outlier("General",0.05);
  };
  R_Date("TKA-20636 NR5A2-6-3 On Floor 13", 2838, 23)
  {
   Outlier("General",0.05);
  };
  Boundary("End Floor 13");
```

```
 };
 Sequence("5B F16-11")
 {
  Boundary("Start Floor 16");
  R_Date("TKa-26472 NR5B4-6-10 Floor 16 fill",2819,21)
  {
   Outlier("General",0.05);
  };
  R_Date("TKa-26471 NR5B2-6-8 Floor 14 fill",2746,21)
  {
   Outlier("General",0.05);
  };
  Boundary("End Floor 14");
 };
};
Boundary("Floor 14-Floor 11");
R_Date("TKa-26469 NR5B2-6-2 Floor 11 fill",2784,20)
{
 Outlier("General",0.05);
};
Boundary("Floor 11-Floor 10");
Phase("Cruciform&on Floor 10")
{
 R_Date("AA-114537  NR5B3-5-4  On Floor 10",2748,52)
 {
  Outlier("General",0.05);
 };
 Sequence("Cruciform")
 {
  Boundary();
  //Phase("Cache NR11"){
  //R_Date("TKA-28082 (NR5B8-5-28)",3019,26){Outlier("General",0.05);};
  R_Date("TKA-28028 (NR5B8-5-28)",2793,21)
  {
   Outlier("General",0.05);
  };
  //};
  Boundary();
  Phase("Cruciform fill")
  {
   R_Date("TKA-28024 (NR5B8-5-6)",2714,20)
   {
    Outlier("General",0.05);
   };
   R_Date("TKA-28025 (NR5B8-5-10)",2711,20)
   {
    Outlier("General",0.05);
   };
   R_Date("TKA-28026 (NR5B8-5-10)",2710,20)
   {
    Outlier("General",0.05);
```

```
    };
    //R_Date("TKA-28027 (NR5B14-5-11)",2859,21){Outlier("General",0.05);};
   };
   Boundary();
  };
 };
 Boundary("Floor 10-Floor 9");
 Phase("Floor 9")
 {
  R_Date("TKa-26482 NR5B5-5-7 Floor 9 fill",2752,21)
  {
   Outlier("General",0.05);
  };
  R_Date("TKa-26483 NR5B6-5-2 Floor 9 fill",2729,20)
  {
   Outlier("General",0.05);
  };
  R_Date("TKA-28019 (NR5B10-5-4)",2662,21)
  {
   Outlier("General",0.05);
  };
  R_Date("AA-114529  NR5B1-5-2  On Floor 9/fill",2768,26)
  {
   Outlier("General",0.05);
  };
 };
 Boundary("Floor 9-Caches");
 Phase("Cache NR6,7,8,9")
 {
  Sequence("Floors 9-6")
  {
   Boundary("Floor 8-Trench");
   Phase("Trench-Cache NR6")
   {
    Sequence("Trench-Floor6")
    {
     R_Date("TKA-26584 NR5B5-5-10 Trench fill",2697,21)
     {
      Outlier("General",0.05);
     };
     R_Date("AA-114530  NR5B4-4-8  On Floor 6",2499,25)
     {
      Outlier("General",0.05);
     };
    };
    Sequence("S Cache NR6")
    {
     Boundary();
     Phase("Cache NR6")
     {
      R_Date("TKa-26473 NR5B5-5-4 Cache NR6",2742,20)
```

```
    {
     Outlier("General",0.05);
    };
    R_Date("TKa-26476 NR5B5-5-4 Cache NR6",2705,21)
    {
     Outlier("General",0.05);
    };
   };
   Boundary();
  };
 };
 Boundary();
};
Sequence()
{
 Boundary();
 Phase("Caches NR7,8,9")
 {
  Sequence("s Cahce NR7")
  {
   Boundary();
   Phase("Cache NR7")
   {
    R_Date("TKA-28017 (NR5B8-5-3)",2698,21)
    {
     Outlier("General",0.05);
    };
    R_Date("TKA-28018 (NR5B8-5-3)",2528,21)
    {
     Outlier("General",0.05);
    };
   };
   Boundary();
  };
  Sequence()
  {
   Boundary();
   R_Date("TKA-28020 NR5B10-5-3 Cache NR8",2696,21)
   {
    Outlier("General",0.05);
   };
   Boundary();
   Phase("Cache NR9")
   {
    R_Date("TKA-28021 (NR5B10-5-6)",2752,21)
    {
     Outlier("General",0.05);
    };
    R_Date("TKA-28022 (NR5B10-5-6)",2672,21)
    {
     Outlier("General",0.05);
```

```
        };
        //R_Date("TKA-28023 (NR5B10-5-6)",2811,21){ Outlier("General",0.05);};
       };
       Boundary();
       Before()
       {
        Date("=TKA-26584 NR5B5-5-10 Trench fill");
       };
      };
     };
     Boundary();
     Before()
     {
      Date("=AA-114530  NR5B4-4-8  On Floor 6");
     };
    };
   };
   Boundary("End Floor 6");
  };
  Boundary("End Middle Preclassic NR5A");
  Boundary("Start Late-Terminal Preclassic NR5A");
  R_Date("TKA-20634 NR5A2-4-4 On Floor 4", 2055, 22)
  {
   Outlier("General",0.05);
  };
  Boundary("End Late-Terminal Preclassic NR5A");
 };
};
```

NR5 Charcoal Model

```
Plot()
 {
 Outlier_Model("Charcoal",Exp(1,-10,0),U(0,3),"t");
 Sequence("NR5AB")
 {
  Boundary("Start E-Group Plaza");
  Sequence("Middle Preclassic")
  {
   Boundary("Start Floor 23");
   Phase("Floor 23 fill")
   {
    R_Date("TKA-26589 NR5B3-7-9 black clay",2834,20)
    {
     Outlier("Charcoal",1);
    };
    R_Date("TKA-26590 NR5B3-7-9 black clay",2810,21)
    {
     Outlier("Charcoal",1);
    };
```

```
 R_Date("TKA-20669 NR5A1-7-8 Floor 22 fill", 2834, 20)
 {
  Outlier("Charcoal",1);
 };
 R_Date("TKA-26901 NR5A1-7-9 black clay",2787,20)
 {
  Outlier("Charcoal",1);
 };
};
Boundary("Floor 23-Floor 22");
Phase("Floor 22 fill cuadros")
{
 R_Date("TKA-26588 NR5B1-7-7 earthen fill",2832,21)
 {
  Outlier("Charcoal",1);
 };
 R_Date("TKA-26587 NR5B1-7-6 earthen fill",2814,20)
 {
  Outlier("Charcoal",1);
 };
 R_Date("TKA-26586 NR5B1-7-6 earthen fill",2799,21)
 {
  Outlier("Charcoal",1);
 };
 R_Date("TKA-26583 NR5B3-7-5 Floor 22 fill",2817,21)
 {
  Outlier("Charcoal",1);
 };
 R_Date("TKA-20668 NR5A1-7-5 Under Floor 22", 2805, 21)
 {
  Outlier("Charcoal",1);
 };
};
Boundary("Floor 22-Floor 21");
Phase("Floor 21 fill")
{
 R_Date("TKa-26486 NR5B3-7-4 Floor 21 fill",2760,21)
 {
  Outlier("Charcoal",1);
 };
 R_Date("TKA-26581 NR5B3-7-4 Floor 21 fill",2747,20)
 {
  Outlier("Charcoal",1);
 };
};
Boundary("Floor 21-Floor 20");
Phase("Floor 20 fill")
{
 R_Date("TKa-26484 NR5B1-7-3 Floor 20 fill",2800,21)
 {
  Outlier("Charcoal",1);
```

```
  };
  R_Date("TKa-26485 NR5B1-7-3 Floor 20 fill",2795,21)
  {
   Outlier("Charcoal",1);
  };
 };
 Boundary("Floor 20-Floor 19");
 Phase("Floor 19 fill Cuadros")
 {
  //R_Date("TKA-20638 NR5A1-6-13 Floor 19 fill", 3800, 25){Outlier();};
  R_Date("TKa-26479 NR5B1-7-1 Cuadros",2785,20)
  {
   Outlier("Charcoal",1);
  };
  R_Date("TKa-26478 NR5B1-6-10 Floor 19 fill",2811,21)
  {
   Outlier("Charcoal",1);
  };
 };
 Boundary("Floor 19-Floor 18");
 R_Date("TKa-26477 NR5B3-6-9 On Floor 19",2755,20)
 {
  Outlier("Charcoal",1);
 };
 Boundary("Floor 18-Floor 16");
 Phase("Floor 18-Floor 11")
 {
  Sequence("5A F14-13")
  {
   Boundary("Start Floor 14");
   R_Date("TKA-20637 NR5A1-6-6 On Floor 16", 2845, 23)
   {
    Outlier("Charcoal",1);
   };
   R_Date("TKA-20635 NR5A2-6-4 On Floor 14", 2835, 25)
   {
    Outlier("Charcoal",1);
   };
   R_Date("TKA-20636 NR5A2-6-3 On Floor 13", 2838, 23)
   {
    Outlier("Charcoal",1);
   };
   Boundary("End Floor 13");
  };
  Sequence("5B F16-11")
  {
   Boundary("Start Floor 16");
   R_Date("TKa-26472 NR5B4-6-10 Floor 16 fill",2819,21)
   {
    Outlier("Charcoal",1);
   };
```

```
  R_Date("TKa-26471 NR5B2-6-8 Floor 14 fill",2746,21)
  {
   Outlier("Charcoal",1);
  };
  Boundary("End Floor 14");
 };
};
Boundary("Floor 14-Floor 11");
R_Date("TKa-26469 NR5B2-6-2 Floor 11 fill",2784,20)
{
 Outlier("Charcoal",1);
};
Boundary("Floor 11-Floor 10");
Phase("Cruciform&on Floor 10")
{
 R_Date("AA-114537  NR5B3-5-4  On Floor 10",2748,52)
 {
  Outlier("Charcoal",1);
 };
 Sequence("Cruciform")
 {
  Boundary();
  //Phase("Cache NR11"){
  //R_Date("TKA-28082 (NR5B8-5-28)",3019,26){Outlier();};
  R_Date("TKA-28028 (NR5B8-5-28)",2793,21)
  {
   Outlier("Charcoal",1);
  };
  //};
  Boundary();
  Phase("Cruciform fill")
  {
   R_Date("TKA-28024 (NR5B8-5-6)",2714,20)
   {
    Outlier("Charcoal",1);
   };
   R_Date("TKA-28025 (NR5B8-5-10)",2711,20)
   {
    Outlier("Charcoal",1);
   };
   R_Date("TKA-28026 (NR5B8-5-10)",2710,20)
   {
    Outlier("Charcoal",1);
   };
   //R_Date("TKA-28027 (NR5B14-5-11)",2859,21){Outlier();};
  };
  Boundary();
 };
};
Boundary("Floor 10-Floor 9");
Phase("Floor 9")
```

```
{
 R_Date("TKa-26482 NR5B5-5-7 Floor 9 fill",2752,21)
 {
  Outlier("Charcoal",1);
 };
 R_Date("TKa-26483 NR5B6-5-2 Floor 9 fill",2729,20)
 {
  Outlier("Charcoal",1);
 };
 R_Date("TKA-28019 (NR5B10-5-4)",2662,21)
 {
  Outlier("Charcoal",1);
 };
 R_Date("AA-114529  NR5B1-5-2  On Floor 9/fill",2768,26)
 {
  Outlier("Charcoal",1);
 };
};
Boundary("Floor 9-Caches");
Phase("Cache NR6,7,8,9")
{
 Sequence("Floors 9-6")
 {
  Boundary("Floor 8-Trench");
  Phase("Trench-Cache NR6")
  {
   Sequence("Trench-Floor6")
   {
    R_Date("TKA-26584 NR5B5-5-10 Trench fill",2697,21)
    {
     Outlier("Charcoal",1);
    };
    R_Date("AA-114530  NR5B4-4-8  On Floor 6",2499,25)
    {
     Outlier("Charcoal",1);
    };
   };
   Sequence("S Cache NR6")
   {
    Boundary();
    Phase("Cache NR6")
    {
     R_Date("TKa-26473 NR5B5-5-4 Cache NR6",2742,20)
     {
      Outlier("Charcoal",1);
     };
     R_Date("TKa-26476 NR5B5-5-4 Cache NR6",2705,21)
     {
      Outlier("Charcoal",1);
     };
    };
```

```
   Boundary();
  };
 };
 Boundary();
};
Sequence()
{
 Boundary();
 Phase("Caches NR7,8,9")
 {
  Sequence("s Cahce NR7")
  {
   Boundary();
   Phase("Cache NR7")
   {
    R_Date("TKA-28017 (NR5B8-5-3)",2698,21)
    {
     Outlier("Charcoal",1);
    };
    R_Date("TKA-28018 (NR5B8-5-3)",2528,21)
    {
     Outlier("Charcoal",1);
    };
   };
   Boundary();
  };
  Sequence()
  {
   Boundary();
   R_Date("TKA-28020 NR5B10-5-3 Cache NR8",2696,21)
   {
    Outlier("Charcoal",1);
   };
   Boundary();
   Phase("Cache NR9")
   {
    R_Date("TKA-28021 (NR5B10-5-6)",2752,21)
    {
     Outlier("Charcoal",1);
    };
    R_Date("TKA-28022 (NR5B10-5-6)",2672,21)
    {
     Outlier("Charcoal",1);
    };
    //R_Date("TKA-28023 (NR5B10-5-6)",2811,21){ Outlier();};
   };
   Boundary();
   Before()
   {
    Date("=TKA-26584 NR5B5-5-10 Trench fill");
   };
```

```
     };
     };
     Boundary();
     Before()
     {
      Date("=AA-114530  NR5B4-4-8  On Floor 6");
     };
    };
   };
   Boundary("End Floor 6");
  };
  Boundary("End Middle Preclassic NR5A");
  Boundary("Start Late-Terminal Preclassic NR5A");
  R_Date("TKA-20634 NR5A2-4-4 On Floor 4", 2055, 22)
  {
   Outlier("Charcoal",1);
  };
  Boundary("End Late-Terminal Preclassic NR5A");
 };
};
```

LN1C Final Model

```
Plot()
 {
  Sequence("LN1C")
  {
   Boundary("Start LN1C");
   Phase("Floors 5-3")
   {
    R_Date("TKA-28031 (LN1C5-4-9)",2714,20);
    R_Date("TKA-28034 (LN1C3-4-10)",2709,20);
    Sequence("sU7&8 midden")
    {
     Boundary();
     Phase("U7&8 midden")
     {
      R_Date("TKA-28032 (LN1C7-4-8)",2712,20);
      R_Date("TKA-28033 (LN1C6-4-8)",2703,20);
     };
     Boundary();
    };
    Sequence("U2")
    {
     Boundary();
     R_Date("TKA-28029 (LN1C2-4-7)",2780,21);
     Boundary();
     Phase("U2 midden")
     {
      R_Date("TKA-28035 (LN1C2-4-5)",2756,20);
```

```
      R_Date("TKA-28036 (LN1C2-4-5)",2740,20);
     };
     Boundary();
    };
   };
  Boundary("End LN1C");
 };
};
```

LN1C General Outlier Model

```
Plot()
 {
  Outlier_Model("General",T(5),U(0,4),"t");
  Sequence("LN1C")
  {
   Boundary();
   Phase("Floors 5-3")
   {
    R_Date("TKA-28031 (LN1C5-4-9)",2714,20)
    {
     Outlier("General",0.05);
    };
    R_Date("TKA-28034 (LN1C3-4-10)",2709,20)
    {
     Outlier("General",0.05);
    };
    Sequence("sU7&8 midden")
    {
     Boundary();
     Phase("U7&8 midden")
     {
      R_Date("TKA-28032 (LN1C7-4-8)",2712,20)
      {
       Outlier("General",0.05);
      };
      R_Date("TKA-28033 (LN1C6-4-8)",2703,20)
      {
       Outlier("General",0.05);
      };
     };
     Boundary();
    };
    Sequence("U2")
    {
     Boundary();
     Phase("U2 lower")
     {
      R_Date("TKA-28029 (LN1C2-4-7)",2780,21)
```

```
    {
     Outlier("General",0.05);
    };
    R_Date("TKA-28030 (LN1C2-4-7)",2829,21)
    {
     Outlier("General",0.05);
    };
   };
   Boundary();
   Phase("U2 midden")
   {
    R_Date("TKA-28035 (LN1C2-4-5)",2756,20)
    {
     Outlier("General",0.05);
    };
    R_Date("TKA-28036 (LN1C2-4-5)",2740,20)
    {
     Outlier("General",0.05);
    };
   };
   Boundary();
  };
 };
 Boundary();
};
};
```

REFERENCES AND NOTES

1. O. Dietrich, The role of cult and feasting in the emergence of Neolithic communities: New evidence from Göbekli Tepe, south-eastern Turkey. *Antiquity* **86**, 674–695 (2012).

2. R. Shady Solis, J. Haas, W. Creamer, Dating Caral, a preceramic site in the Supe Valley on the Central Coast of Peru. *Science* **292**, 723–726 (2001).

3. T. R. Kidder, in *Hunter-Gatherer Archaeology as Historical Process*, K. E. Sassaman, D. H. Holly, Eds. (Univ. of Arizona Press, 2011), pp. 95–119.

4. D. Graeber, D. Wengrow, *The Dawn of Everything: A New History of Humanity* (Farrar, Straus, and Giroux, 2021).

5. K. E. Sassaman, Complex hunter-gatherers in evolution and history: A North American perspective. *J. Archaeol. Res.* **12**, 227–280 (2004).

6. C. Stanish, *The Evolution of Human Co-operation: Ritual and Social Complexity in Stateless Societies* (Cambridge Univ. Press, 2017).

7. R. L. Burger, R. M. Rosenswig, Eds., *Early New World Monumentality* (Univ. Press of Florida, 2012), pp. 198–230.

8. R. E. W. Adams, Ed., *The Origins of Maya Civilization* (Univ. of New Mexico Press, 1977).

9. G. R. Willey, *Excavations at Seibal, Department of Peten, Guatemala: General Summary and Conclusions*, Memoirs of the Peabody Museum of Archaeology and Ethnology (Harvard University, 1990), vol. 17, no. 4.

10. T. Inomata, D. Triadan, J. MacLellan, M. Burham, K. Aoyama, J. M. Palomo, H. Yonenobu, F. Pinzón, H. Nasu, High-precision radiocarbon dating of political collapse and dynastic origins at the Maya site of Ceibal, Guatemala. *Proc. Natl. Acad. Sci. U.S.A.* **114**, 1293–1298 (2017).

11. T. Inomata, J. MacLellan, D. Triadan, J. Munson, M. Burham, K. Aoyama, H. Nasu, F. Pinzon, H. Yonenobu, Development of sedentary communities in the Maya lowlands:

Coexisting mobile groups and public ceremonies at Ceibal, Guatemala. *Proc. Natl. Acad. Sci. U.S.A.* **112**, 4268–4273 (2015).

12. T. Inomata, D. Triadan, K. Aoyama, V. Castillo, H. Yonenobu, Early ceremonial constructions at Ceibal, Guatemala, and the origins of lowland Maya civilization. *Science* **340**, 467–471 (2013).

13. T. Inomata, D. Triadan, F. Pinzón, K. Aoyama, Artificial plateau construction during the preclassic period at the Maya site of Ceibal, Guatemala. *PLOS ONE* **14**, e0221943 (2019).

14. M. K. Brown, G. J. Bey, Eds., *Pathways to Complexity: A View from the Maya Lowlands* (Univ. Press of Florida, 2018).

15. D. A. Freidel, A. F. Chase, A. S. Dowd, J. Murdock, Eds., *Maya E Groups: Calendars, Astronomy, and Urbanism in the Early Lowlands* (Univ. Press of Florida, 2017).

16. F. Estrada-Belli, *The First Maya Civilization: Ritual and Power Before the Classic Period* (Routledge, 2011), pp. 176.

17. D. Freidel, A. F. Chase, A. S. Dowd, J. Murdock, Eds., *The Materialization of Time in the Ancient Maya World: Mythic History and Ritual Order* (Univ. Press of Florida, 2024).

18. T. Inomata, D. Triadan, V. A. Vázquez López, J. C. Fernandez-Diaz, T. Omori, M. B. Méndez Bauer, M. García Hernández, T. Beach, C. Cagnato, K. Aoyama, H. Nasu, Monumental architecture at Aguada Fénix and the rise of Maya civilization. *Nature* **582**, 530–533 (2020).

19. J. E. Clark, in *The Origins of Maya States*, L. P. Traxler, R. J. Sharer, Eds. (University of Pennsylvania Museum of Archaeology and Anthropology, 2016), pp. 123–224.

20. G. W. Lowe, in *The Origins of Maya Civilization*, R. E. W. Adams, Ed. (Univ. of New Mexico Press, 1977), pp. 197–248.

21. D. Triadan, V. Castillo, T. Inomata, J. M. Palomo, M. B. Méndez, M. Cortave, J. MacLellan, M. Burham, E. Ponciano, Social transformation in a Middle Preclassic community: Elite residential complexes at Ceibal. *Anc. Mesoam.* **28**, 233–264 (2017).

22. F. Estrada-Belli, Lightning sky, rain, and the Maize God: The ideology of Preclassic Maya rulers at Cival, Peten, Guatemala. *Anc. Mesoam.* **17**, 57–78 (2006).

23. G. W. Lowe, in *The Olmec and Their Neighbors*, M. D. Coe, D. Grove, Eds. (Dumbarton Oaks Research Library and Collection, 1981), pp. 231–256.

24. B. R. Bachand, L. S. Lowe, in *Arqueología Reciente de Chiapas: Contribuciones del Encuentro Celebrado en el 600 Aniversario de la Fundación Arqueológica Nuevo Mundo*, L. S. Lowe, M. E. Pye, Eds. (Brigham Young University, 2012), pp. 45–68. [Recent Archaeology of Chiapas: Contributions of the Meeting Celebrating the 60th Anniversary of the New World Archaeological Foundation].

25. T. Inomata, J. C. Fernandez-Diaz, D. Triadan, M. García Mollinedo, F. Pinzón, M. García Hernández, A. Flores, A. Sharpe, T. Beach, G. W. L. Hodgins, J. J. Durón Díaz, A. Guerra Luna, L. Guerrero Chávez, M. d. L. Hernández Jiménez, M. Moreno Díaz, Origins and spread of formal ceremonial complexes in the Olmec and Maya regions revealed by airborne lidar. *Nat. Hum. Behav.* **5**, 1487–1501 (2021).

26. I. Šprajc, T. Inomata, A. F. Aveni, Origins of Mesoamerican astronomy and calendar: Evidence from the Olmec and Maya regions. *Sci. Adv.* **9**, eabq7675 (2023).

27. I. Šprajc, T. Inomata, Astronomy, architecture, and landscape in the Olmec Area and Western Maya Lowlands: Implications for understanding regional variability and evolution of orientation patterns in Mesoamerica. *Lat. Am. Antiq.* **35**, 381–401 (2024).

28. T. W. Pugh, P. M. Rice, E. M. C. Nieto, M. L. Meranda, D. S. Milley, Middle Preclassic hydraulic planning at Nixtun-Ch'ich', Peten, Guatemala. *Anc. Mesoam.* **33**, 589–603 (2022).

29. T. W. Pugh, P. M. Rice, Early urban planning, spatial strategies, and the Maya gridded city of Nixtun-Ch'ich', Petén, Guatemala. *Curr. Anthropol.* **58**, 576–603 (2017).

30. T. Omori, K. Yamada, I. Kitaba, T. Hori, T. Nakagawa, Reliable radiocarbon dating of fossil pollen grains: It is truly possible. *Quat. Geochronol.* **77**, 101456 (2023).

31. K. Yamada, T. Omori, I. Kitaba, T. Hori, T. Nakagawa, Extraction method for fossil pollen grains using a cell sorter suitable for routine ^{14}C dating. *Quat. Geochronol.* **272**, 107236 (2021).

32. S. Houston, C. Brittenham, C. Mesick, A. Tokovinine, C. Warinner, *Veiled Brightness: A History of Ancient Maya Color* (Univ. of Texas Press, 2009).

33. D. Magaloni Kerpel, in *La Pintura Mural Prehispánica en México II: Área Maya, Bonampak, Tomo 2*, B. de la Fuente, Ed. (Universidad Nacional Autónoma de México, Instituto de Investigaciones Estéticas, 1998), pp. 49–80. [Prehispanic Mural Paintings in Mexico II: Maya Area, Bonampak, vol. 2].

34. D. Magaloni Kerpel, in *La Pintura Mural Prehispánica en México I: Teotihuacán, Tomo 2*, B. de la Fuente, Ed. (Universidad Nacional Autónoma de México, Instituto de Investigaciones Estéticas, 1995), pp. 187–225. [Prehispanic Mural Paintings in Mexico I: Teotihuacan, vol. 2].

35. P. Drucker, R. F. Heizer, R. H. Squier, *Excavations at La Venta, Tabasco, 1955*, Bureau of American Ethnology Bulletin (Smithsonian Institution, 1959), vol. 170.

36. D. C. Grove, *The Olmec Paintings of Oxtotitlan Cave, Guerrero, Mexico* (Dumbarton Oaks, 1970).

37. D. Hosler, *The Sounds and Colors of Power: The Sacred Metallurgical Technology of Ancient West Mexico* (MIT Press, 1994).

38. A. González Cruz, *La Reina Roja: Una Tumba Real de Palenque* (INAH, 2011). [Red Queen: A Royal Tomb of Palenque].

39. J. L. Meanwell, E. H. Paris, R. L. Bravo, Material composition of greenstone acquisition and use in the Jovel Valley, Chiapas, Mexico. *Anc. Mesoam.* **36**, 1–25 (2025).

40. J. M. Weeks, in *Archaeometallurgy in Mesoamerica: Current Approaches and New Perspectives*, S. E. Simmons, A. N. Shugar, Eds. (Univ. Press of Colorado, 2013), pp. 113–133.

41. R. J. Roberts, E. M. Irving, *Mineral Deposits of Central America*, Geological Survey Bulletin 1034 (U.S. Government Printing Office, 1957).

42. A. M. Tozzer, *Landa's Relación de las Cosas de Yucatan* (Harvard University, 1941), vol. 18, 394 pp. [Landa's Account of the Things of Yucatan].

43. K. W. Anderson, C. Helmke, The personifications of celestial water: The many guises of the Storm god in the pantheon and cosmology of Teotihuacan. *Contrib. New World Archaeol.* **5**, 165–196 (2013).

44. R. E. MacLaury, *Color and Cognition in Mesoamerica: Constructing Categories as Vantages* (Univ. of Texas Press, 1997).

45. L. López Luján, X. Chávez Balderas, B. Zúñiga-Arellano, A. Aguirre Molina, N. Valentín Maldonado, Un portal al inframundo: Ofrendas de animales sepultadas al pie del Templo Mayor de Tenochtitlan. *Estud. Cult. Náhuatl* **44**, 9–40 (2012). [A Portal to the Underworld: Offerings of Animals Buried at the foot of the Great Temple of Tenochtitlan].

46. M. D. Coe, A model of ancient community structure in the Maya Lowlands. *Southwest. J. Anthropol.* **21**, 97–114 (1965).

47. W. M. Ringle, in *Social Patterns in Pre-classic Mesoamerica*, D. C. Grove, R. A. Joyce, Eds. (Dumbarton Oaks, 1999), pp. 183–223.

48. W. Ashmore, Site-planning principles and concepts of directionality among the ancient Maya. *Lat. Am. Antiq.* **2**, 199–226 (1991).

49. W. Ashmore, J. A. Sabloff, Spatial orders in Maya civic plans. *Lat. Am. Antiq.* **13**, 201–215 (2002).

50. S. R. Hutson, J. A. Welch, Roadwork: Long-distance causeways at Uci, Yucatan, Mexico. *Lat. Am. Antiq.* **32**, 310–330 (2021).

51. D. A. Freidel, J. A. Sabloff, *Cozumel: Late Maya Settlement Patterns* (Academic Press, 1984).

52. E. Z. Vogt, *Tortillas for the Gods: A Symbolic Analysis of Zinacanteco Rituals* (Harvard Univ. Press, 1976).

53. G. H. Gossen, *Chamulas in the World of the Sun: Time and Space in a Maya Oral Tradition* (Harvard Univ. Press, 1974).

54. M. D. Coe, R. A. Diehl, *In the Land of the Olmec* (Univ. of Texas Press, 1980).

55. V. L. Scarborough, *The Flow of Power: Ancient Water Systems and Landscapes* (SAR Press, 2003).

56. L. J. Lucero, *Water and Ritual: The Rise and Fall of Classic Maya Rulers* (Univ. of Texas Press, 2006).

57. A. Cyphers, *El Palacio Rojo: Herencia de Gobernantes Olmecas* (Universidad Nacional Autónoma de México, 2021). [Red Palace: Legacy of Olmec Rulers].

58. L. I. Paradis, in *Early New World Monumentality*, R. L. Burger, R. M. Rosenswig, Eds. (Univ. Press of Florida, 2012), pp. 174–197.

59. E. Harrison-Buck, S. M. Krause, M. Brouwer Burg, M. Willis, A. Perrotti, K. Bailey, Late Archaic large-scale fisheries in the wetlands of the pre-Columbian Maya Lowlands. *Sci. Adv.* **10**, eadq1444 (2024).

60. J. A. Neely, M. J. Aiuvalasit, V. A. Clause, New light on the prehistoric Purrón Dam Complex: Small corporate group collaboration in the Tehuacán Valley, Puebla, México. *J. Field Archaeol.* **40**, 347–364 (2015).

61. C. L. Erickson, An artificial landscape-scale fishery in the Bolivian Amazon. *Nature* **408**, 190–193 (2000).

62. T. Inomata, D. Triadan, K. Aoyama, After 40 years: Revisiting Ceibal to investigate the origins of lowland Maya civilization. *Anc. Mesoam.* **28**, 187–201 (2017).

63. C. Bronk Ramsey, OxCal 4.4 (2024); http://c14.arch.ox.ac.uk/.

64. C. Bronk Ramsey, Bayesian analysis of radiocarbon dates. *Radiocarbon* **51**, 337–360 (2009).

65. P. J. Reimer, W. E. Austin, E. Bard, A. Bayliss, P. G. Blackwell, C. B. Ramsey, M. Butzin, H. Cheng, R. L. Edwards, M. Friedrich, The IntCal20 Northern Hemisphere radiocarbon age calibration curve (0–55 cal kBP). *Radiocarbon* **62**, 725–757 (2020).

66. C. Bronk Ramsey, Radiocarbon calibration and analysis of stratigraphy: The OxCal program. *Radiocarbon* **37**, 425–430 (1995).

67. C. Bronk Ramsey, Analysis of chronological information and radiocarbon calibration: The program OxCal. *Archaeol. Comput. Newsl.* **41**, 11–16 (1994).

68. C. E. Buck, W. G. Cavanagh, C. D. Litton, *Bayesian Approach to Interpreting Archaeological Data* (Wiley, 1996).

69. A. Bayliss, Quality in Bayesian chronological models in archaeology. *World Archaeol.* **47**, 677–700 (2015).

70. W. D. Hamilton, A. M. Krus, The myths and realities of Bayesian chronological modeling revealed. *Am. Antiq.* **83**, 187–203 (2018).

71. B. Arroyo, T. Inomata, G. Ajú, J. Estrada, H. Nasu, K. Aoyama, Refining Kaminaljuyu chronology: New radiocarbon dates, Bayesian analysis, and ceramics studies. *Lat. Am. Antiq.* **31**, 477–497 (2020).

72. T. Inomata, R. Ortiz, B. Arroyo, E. J. Robinson, Chronological revision of Preclassic Kaminaljuyú, Guatemala: Implications for social processes in the Southern Maya area. *Lat. Am. Antiq.* **25**, 377–408 (2014).

73. T. Inomata, in *Pre-Mamom Pottery Variation and the Preclassic Origins of the Lowland Maya*, D. Walker, Ed. (Univ. Press of Colorado, 2023), pp. 167–203.

74. C. Bronk Ramsey, Dealing with outliers and offsets in radiocarbon dating. *Radiocarbon* **51**, 1023–1045 (2009).

75. M. Dee, C. Bronk Ramsey, High-precision Bayesian modeling of samples susceptible to inbuilt age. *Radiocarbon* **56**, 83–94 (2013).

76. R. Millon, *Urbanization at Teotihuacán, Mexico, Volume 1: The Teotihuacan Map, Part 1* (Univ. of Texas Press, 1973).

77. R. F. Carr, J. E. Hazard, *Tikal Report 11: Map of the Ruins of Tikal, El Petén, Guatemala* (University of Pennsylvania Museum of Archaeology and Anthropology, 1961).

78. N. Sugiyama, S. Sugiyama, S. Alejandro, Inside the Sun Pyramid at Teotihuacan, Mexico: 2008-2011 excavations and preliminary results. *Lat. Am. Antiq.* **24**, 403–432 (2013).

79. L. Beramendi-Orosco, G. Gonzalez-Hernandez, J. Urrutia-Fucugauchi, L. Manzanilla, A. Soler-Arechalde, A. Goguitchaishvili, N. Jarboe, High-resolution chronology for the Mesoamerican urban center of Teotihuacan derived from Bayesian statistics of radiocarbon and archaeological data. *Quatern. Res.* **71**, 99–107 (2009).

80. S. Martin, N. Grube, *Chronicle of the Maya Kings and Queens: Deciphering the Dynasties of the Ancient Maya* (Thames & Hudson, ed. 2, 2008), 240 pp.

81. A. F. Chase, D. Z. Chase, J. J. Awe, J. F. Weishampel, G. Iannone, H. Moyes, J. Yaeger, M. K. Brown, R. L. Shrestha, W. E. Carter, Ancient Maya regional settlement and inter-site analysis: The 2013 west-central Belize LiDAR survey. *Remote Sens.* **6**, 8671–8695 (2014).

82. A. F. Chase, D. Z. Chase, C. T. Fisher, S. J. Leisz, J. F. Weishampel, Geospatial revolution and remote sensing LiDAR in Mesoamerican archaeology. *Proc. Natl. Acad. Sci. U.S.A.* **109**, 12916–12921 (2012).

83. A. F. Chase, D. Z. Chase, J. F. Weishampel, J. B. Drake, R. L. Shrestha, K. C. Slatton, J. J. Awe, W. E. Carter, Airborne LiDAR, archaeology, and the ancient Maya landscape at Caracol, Belize. *J. Archaeol. Sci.* **38**, 387–398 (2011).

84. M. A. Canuto, F. Estrada-Belli, T. G. Garrison, S. D. Houston, M. J. Acuña, M. Kovac, D. Marken, P. Nondédéo, L. Auld-Thomas, C. Castanet, D. Chatelain, C. R. Chiriboga, T. Å. Drápela, T. Lieskovský, A. Tokovinine, A. Velasquez, J. C. Fernandez-Diaz, R. Shrestha,

Ancient lowland Maya complexity as revealed by airborne laser scanning of northern Guatemala. *Science* **361**, eaau0137 (2018).

85. S. R. Hutson, B. Kidder, C. Lamb, D. Vallejo-Cáliz, J. Welch, Small buildings and small budgets: Making Lidar work in northern Yucatan, Mexico. *Adv. Archaeol. Pract.* **4**, 268–283 (2016).

86. T. Hare, M. Masson, B. Russell, High-density LiDAR mapping of the ancient city of Mayapán. *Remote Sens.* **6**, 9064–9085 (2014).

87. N. Sugiyama, S. Sugiyama, T. Catignani, A. S. Chase, J. C. Fernandez-Diaz, Humans as geomorphic agents: Lidar detection of the past, present and future of the Teotihuacan Valley, Mexico. *PLOS ONE* **16**, e0257550 (2021).

88. C. T. Fisher, J. C. Fernández-Diaz, A. S. Cohen, O. N. Cruz, A. M. Gonzáles, S. J. Leisz, F. Pezzutti, R. Shrestha, W. Carter, Identifying ancient settlement patterns through LiDAR in the Mosquitia region of Honduras. *PLOS ONE* **11**, e0159890 (2016).

89. C. Golden, A. K. Scherer, W. Schroder, T. Murtha, S. Morell-Hart, J. C. Fernandez Diaz, S. D. P. Jiménez Álvarez, O. Alcover Firpi, M. Agostini, A. Bazarsky, Airborne Lidar survey, density-based clustering, and ancient Maya settlement in the Upper Usumacinta River Region of Mexico and Guatemala. *Remote Sens.* **13**, 4109 (2021).

90. T. W. Stanton, T. Ardren, N. C. Barth, J. C. Fernandez-Diaz, P. Rohrer, D. Meyer, S. J. Miller, A. Magnoni, M. Pérez, 'Structure' density, area, and volume as complementary tools to understand Maya Settlement: An analysis of lidar data along the great road between Coba and Yaxuna. *J. Archaeol. Sci. Rep.* **29**, 102178 (2020).

91. J. C. Fernandez-Diaz, W. E. Carter, C. Glennie, R. L. Shrestha, Z. Pan, N. Ekhtari, A. Singhania, D. Hauser, M. Sartori, Capability assessment and performance metrics for the Titan multispectral mapping lidar. *Remote Sens.* **8**, 936 (2016).

92. J. C. Fernandez-Diaz, W. E. Carter, R. L. Shrestha, C. L. Glennie, Now you see it… now you don't: Understanding airborne mapping LiDAR collection and data product generation for archaeological research in Mesoamerica. *Remote Sens.* **6**, 9951–10001 (2014).

93. R. Bennett, K. Welham, A. Ford, A comparison of visualization techniques for models created from airborne laser scanned data. *Archaeol. Prospect.* **19**, 41–48 (2012).

94. K. Challis, P. Forlin, M. Kincey, A generic toolkit for the visualization of archaeological features on airborne LiDAR elevation data. *Archaeol. Prospect.* **18**, 279–289 (2011).

95. B. J. Devereux, G. S. Amable, P. Crow, Visualisation of LiDAR terrain models for archaeological feature detection. *Antiquity* **82**, 470–479 (2008).

96. B. Štular, E. Lozić, S. Eichert, Airborne LiDAR-derived digital elevation model for archaeology. *Remote Sens.* **13**, 1855 (2021).

97. B. Štular, Ž. Kokalj, K. Oštir, L. Nuninger, Visualization of lidar-derived relief models for detection of archaeological features. *J. Archaeol. Sci.* **39**, 3354–3360 (2012).

98. T. Chiba, S.-i. Kaneta, Y. Suzuki, Red relief image map: New visualization method for three dimensional data. *Int. Arch. Photogramm. Remote Sens. Spat. Inf. Sci.* **37**, 1071–1076 (2008).

99. J. A. Sabloff, *Excavations at Seibal, Department of Peten, Guatemala: Ceramics*, Memoirs of the Peabody Museum of Archaeology and Ethnology (Harvard University, 1975), vol. 13, no. 2.

100. T. Inomata, The emergence of standardized spatial plans in southern Mesoamerica: Chronology and interregional interactions viewed from Ceibal, Guatemala. *Anc. Mesoam.* **28**, 329–355 (2017).

101. R. J. Gettens, E. W. Fitzhugh, Malachite and green verditer. *Stud. Conserv.* **19**, 2–23 (1974).

102. R. J. Gettens, E. W. Fitzhugh, Azurite and blue verditer. *Stud. Conserv.* **11**, 54–61 (1966).

103. N. Eastaugh, V. Walsh, T. Chaplin, R. Siddall, *Pigment Compendium: A Dictionary and Optical Microscopy of Historic Pigments* (Routledge, 2008).

104. D. R. Piperno, *Phytoliths: A comprehensive Guide for Archaeologists and Paleoecologists* (AltaMira Press, 2006).

105. C. Bonomelli, V. Fernández, F. Capurro, C. Palma, X. Videla, X. Rojas-Silva, A. Nario, J. Mártiz, Absorption and distribution of calcium (^{45}Ca) applied to the surface of orange (*Citrus sinensis*) fruits at different developmental stages. *Agronomy* **12**, 150 (2022).

106. V. R. Franceschi, P. A. Nakata, Calcium oxalate in plants: Formation and function. *Annu. Rev. Plant Biol.* **56**, 41–71 (2005).

107. C. J. Erasmus, Monument building: Some field experiments. *Southwest. J. Anthropol.* **21**, 277–301 (1965).

108. E. M. Abrams, *How the Maya Built Their World: Energetics and Ancient Architecture* (Univ. of Texas Press, 1994).

109. E. M. Abrams, Economic specialization and construction personnel in Classic period Copan, Honduras. *Am. Antiq.* **52**, 485–499 (1987).

110. D. Webster, J. Kirker, Too many Maya, too few buildings: Investigating construction potential at Copán, Honduras. *J. Anthropol. Res.* **51**, 363–387 (1995).

111. A. L. Ortmann, T. R. Kidder, Building Mound A at Poverty Point, Louisiana: Monumental public architecture, ritual practice, and implications for hunter-gatherer complexity. *Geoarchaeology* **28**, 66–86 (2013).

112. T. Beach, S. Luzzadder-Beach, D. Cook, N. Dunning, D. J. Kennett, S. Krause, R. Terry, D. Trein, F. Valdez, Ancient Maya impacts on the Earth's surface: An early anthropocene analog? *Quat. Sci. Rev.* **124**, 1–30 (2015).

113. S. Porder, S. Ramachandran, The phosphorus concentration of common rocks—A potential driver of ecosystem P status. *Plant Soil* **367**, 41–55 (2013).
